# Adolescent binge drinking disrupts normal trajectories of brain functional organization and personality maturation

**DOI:** 10.1016/j.nicl.2019.101804

**Published:** 2019-03-31

**Authors:** Hongtao Ruan, Yunyi Zhou, Qiang Luo, Gabriel H. Robert, Sylvane Desrivières, Erin Burke Quinlan, ZhaoWen Liu, Tobias Banaschewski, Arun L.W. Bokde, Uli Bromberg, Christian Büchel, Herta Flor, Vincent Frouin, Hugh Garavan, Penny Gowland, Andreas Heinz, Bernd Ittermann, Jean-Luc Martinot, Marie-Laure Paillère Martinot, Frauke Nees, Dimitri Papadopoulos Orfanos, Luise Poustka, Sarah Hohmann, Juliane H. Fröhner, Michael N. Smolka, Henrik Walter, Robert Whelan, Fei Li, Gunter Schumann, Jianfeng Feng, IMAGEN Consortium

**Affiliations:** a School of Mathematical Sciences, Fudan University, Shanghai 200433, PR China; b Institute of Science and Technology for Brain-Inspired Intelligence, Fudan University, Shanghai 200433, PR China; c Department of Psychology and the Behavioural and Clinical Neuroscience Institute, University of Cambridge, Cambridge CB2 3EB, UK; d EA 4712 “Behavior and Basal Ganglia”, Rennes University 1, Rennes, France; e Centre for Population Neuroscience and Stratified Medicine (PONS), Institute of Psychiatry, Psychology and Neuroscience, Social, Genetic and Developmental Psychiatry Centre, King's College London, London SE5 8AF, United Kingdom; f School of Computer Science and Technology, Xidian University, Xi'an 710071, Shannxi, PR China; g Department of Child and Adolescent Psychiatry and Psychotherapy, Central Institute of Mental Health, Medical Faculty Mannheim, Heidelberg University, Square J5, 68159 Mannheim, Germany; h Discipline of Psychiatry, School of Medicine and Trinity College Institute of Neuroscience, Trinity College Dublin, Ireland; i University Medical Centre Hamburg-Eppendorf, House W34, 3.OG, Martinistr. 52, 20246, Hamburg, Germany; j Department of Cognitive and Clinical Neuroscience, Central Institute of Mental Health, Medical Faculty Mannheim, Heidelberg University, Square J5, Mannheim, Germany; k Department of Psychology, School of Social Sciences, University of Mannheim, 68131 Mannheim, Germany; l NeuroSpin, CEA, Université Paris-Saclay, F-91191 Gif-sur-Yvette, France; m Departments of Psychiatry and Psychology, University of Vermont, 05405 Burlington, VT, USA; n Sir Peter Mansfield Imaging Centre School of Physics and Astronomy, University of Nottingham, University Park, Nottingham, United Kingdom; o Charité – Universitätsmedizin Berlin, corporate member of Freie Universität Berlin, Humboldt-Universität zu Berlin, Berlin Institute of Health, Department of Psychiatry and Psychotherapy, Campus Charité Mitte, Charitéplatz 1, Berlin, Germany; p Physikalisch-Technische Bundesanstalt (PTB), Braunschweig, Abbestr. 2 - 12, Berlin, Germany; q Institut National de la Santé et de la Recherche Médicale, INSERM Unit 1000 “Neuroimaging & Psychiatry”, University Paris Sud, University Paris Descartes - Sorbonne Paris Cité; and Maison de Solenn, Paris, France; r Institut National de la Santé et de la Recherche Médicale, INSERM Unit 1000 “Neuroimaging & Psychiatry”, University Paris Sud, University Paris Descartes; Sorbonne Université; and AP-HP, Department of Child and Adolescent Psychiatry, Pitié-Salpêtrière Hospital, Paris, France; s Department of Child and Adolescent Psychiatry and Psychotherapy, University Medical Centre Göttingen, von-Siebold-Str. 5, 37075 Göttingen, Germany; t Department of Psychiatry and Neuroimaging Center, Technische Universität Dresden, Dresden, Germany; u School of Psychology and Global Brain Health Institute, Trinity College Dublin, Ireland; v Developmental and Behavioral Pediatric Department & Child Primary Care Department, MOE-Shanghai Key Lab for Children's Environmental Health, Xinhua Hospital Affiliated To Shang Jiaotong University School of Medicine, Shanghai, PR China; w Department of Computer Science, University of Warwick, Coventry, UK; x Collaborative Innovation Center for Brain Science, Fudan University, Shanghai, PR China; y Shanghai Center for Mathematical Sciences, Shanghai, PR China; z Key Laboratory of Computational Neuroscience and Brain-Inspired Intelligence (Fudan University), Ministry of Education, PR China

**Keywords:** Adolescent, Binge drinking, Resting state, Personality, Genome, Co-development, rsfMRI, resting-state fMRI, rsFC, resting-state functional connectivity, SNP, single nucleotide polymorphism, ESPAD, European School Survey Project on Alcohol and Drugs, Cantab, Cambridge Neuropsychological Test Automated Battery, NEO-PI-R, Revised NEO Personality Inventory, SURPS, Substance Use Risk Profile Scale, FDR, false discovery rate, SVM, support-vector machine, iFC, increased rsFC, dFC, decreased rsFC, rSNP, risk SNP, pSNP, protective SNP, LOO, leave-one-out, NRI, net reclassification improvement, ROC, receiver operational characteristic, ANOVA, Analysis of Variance, AUC, area under curve

## Abstract

Adolescent binge drinking has been associated with higher risks for the development of many health problems throughout the lifespan. Adolescents undergo multiple changes that involve the co-development processes of brain, personality and behavior; therefore, certain behavior, such as alcohol consumption, can have disruptive effects on both brain development and personality maturation. However, these effects remain unclear due to the scarcity of longitudinal studies. In the current study, we used multivariate approaches to explore discriminative features in brain functional architecture, personality traits, and genetic variants in 19-year-old individuals (*n* = 212). Taking advantage of a longitudinal design, we selected features that were more drastically altered in drinkers with an earlier onset of binge drinking. With the selected features, we trained a hierarchical model of support vector machines using a training sample (*n* = 139). Using an independent sample (*n* = 73), we tested the model and achieved a classification accuracy of 71.2%. We demonstrated longitudinally that after the onset of binge drinking the developmental trajectory of improvement in impulsivity slowed down. This study identified the disrupting effects of adolescent binge drinking on the developmental trajectories of both brain and personality.

## Highlights

• History of binge drinking could be identified by multivariate features at age 19.
• Adolescent binge drinking disrupted frontal connectivity maturation in the brain.
• Impulsivity improvement slowed down after the onset of adolescent binge drinking.

## 1 Introduction

Adolescence is characterized by significant developments (Caspi et al., 2005; Giedd and Rapoport, 2010), including the functional segregation and integration of different brain networks through a process of modular evolution (Gu et al., 2015) and developmental improvement of some personality traits, such as impulsivity (Harden and Tucker-Drob, 2011) and agreeableness (Klimstra et al., 2009). These developmental processes are influenced by genetic and environmental factors, such as alcohol misuse during adolescence (Brown et al., 2008; Squeglia and Gray, 2016; Squeglia et al., 2014). The incidence of alcohol misuse peaks between the ages of 18–25 (Blanco et al., 2008; Chen and Kandel, 1995) and appears to decline after 26 years of age (Substance Abuse and Mental Health Services Administration, 2018), which aligns to the brain development and personality maturation. Alcohol misuse can disrupt standard developmental trajectories and these effects can persist into adulthood and subsequently increase the risk of alcohol dependence, violence, drunk driving, and other adverse outcomes later in life (Miller et al., 2007). Therefore, delineating the consequences of adolescent alcohol drinking on brain and personality developmental trajectories may provide new insights into alcohol-affected neural and behavioral changes that are responsible for long-term adverse outcomes (McCambridge et al., 2011; Spear, 2018).

Significant processes in functional segregation, such as weakening connectivity between brain systems; and integration, such as strengthening connectivity within brain systems, have highlighted that brain functional connectivity network organization is particularly vulnerable to adolescent alcohol drinking (Gu et al., 2015). However, few longitudinal investigations have measured the functional consequences of alcohol drinking in the adolescent brain. In a previous study of the IMAGEN consortium (Whelan et al., 2014), the authors focused on establishing a profile at an early age to predict the onset of binge drinking in a 2-year follow-up; however, brain functional connectivity was not investigated. Based on previous epidemiological results (Dawson et al., 2008), we hypothesized that drinking-altered functional connectivity in the brain should satisfy the following three conditions: 1) classify binge drinkers from non-binge drinkers and be independent of other features, such as personality, cognition, and genetics; 2) earlier and heavier binge drinking induces more discriminative alterations; we named this the hypothesis of trend; and 3) not associated with other substance use (e.g. smoking, cannabis use) during adolescence.

In this study, we aimed to clarify the consequences of adolescent binge drinking by exploiting the longitudinal design of the IMAGEN study (G Schumann et al., 2010). This study collected neuroimaging, personality traits, drinking behavior, and genetic variant data from a large population. The IMAGEN study recruited healthy individuals; therefore, alcohol misuse was defined as episodes of lifetime drunkenness (i.e. binge drinking) (Whelan et al., 2014). Different from previous studies, the longitudinal design enabled us to stratify the drinkers according to their onset of binge drinking. By identifying groups of extreme drinkers with the longest and the shortest histories of binge drinking in our sample, we were able to select the features that were associated with binge drinking and that also satisfied the hypothesis of trend. With such a hypothesis-guided feature selection, we imposed more constraints on the developmental course of any selected feature. Other substance use (e.g., cannabis) may have a different onset age compared with binge drinking, and thereby require its associated feature to have a different trend. In other words, our procedure could increase the specificity of the feature selection associated with binge drinking. As the selected features satisfy the hypothesis of trend, we expected to see an intermediate disruptive effect of these features in a medium-term history of binge drinking group; therefore, we confirmed our findings using an independent sample (i.e. the medium-term drinkers). Finally, we applied a longitudinal analysis to assess if the developmental trajectories for the selected features were significantly affected by the age of binge drinking onset.

## 2 Materials and methods

### 2.1 Participants

The IMAGEN study recruited a cohort of healthy adolescents at the age of 14. Informed consent was obtained from all subjects and their parents/guardians. The genetic data were collected at baseline, the neuroimaging data were collected at ages 14 and 19, and environmental and behavioral data were collected at ages 14, 16 and 19. The details of the study design and data quality are described in a previous report (G Schumann et al., 2010). In total, 212 participants were included in the current study; a flowchart of the inclusion criterion is presented in eFig. 1.

### 2.2 Measurements

#### 2.2.1 Resting-state functional imaging data

A proportion of the participants in the IMAGEN study underwent resting-state fMRI (rsfMRI). In the IMAGEN study, much less rsfMRI data (*n* = 389) were collected at the base line (i.e. age 14). As rsfMRI got more and more attention, more data (*n* = 1069) were collected at the follow-up stage (i.e. age 19). All fMRI data were preprocessed by the Data Processing Assistant for Resting State fMRI (rfmri.org/DPARSF). For each individual, we calculated the resting-state functional connectivity (rsFC) between each pair of brain regions [19,900 links for 200 atlas-defined brain regions, Craddock 2011 Atlas (Craddock et al., 2012) eTable 3].

#### 2.2.2 Genome-wide genotype data

Details of genetic data have been reported previously (Desrivieres et al., 2015); we have included detailed descriptions in the eMethods. Briefly, we converted all single nucleotide polymorphisms (SNP) into binary variables; no mutations were assigned the number “zero” and mutations were assigned the number “one”.

#### 2.2.3 Substance use

**Fig. 1:**
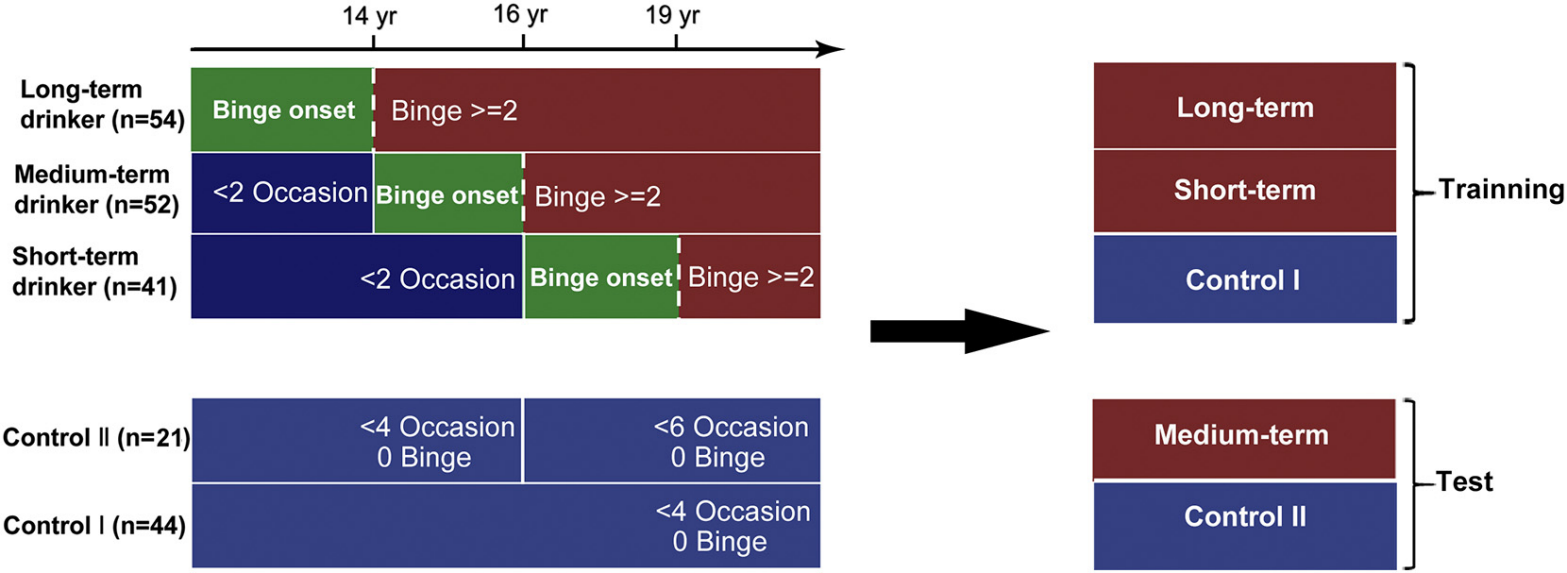
Stratified drinking and control groups. Drinking groups stratified by the onset age of binge drinking. “Binge (ESPAD)” corresponds to the score from the question “on how many occasions in your whole lifetime have you been drunk from drinking alcoholic beverages” while “Occasion (ESPAD)” corresponds to the score from the question “on how many occasions in your whole lifetime have you had any alcoholic beverage to drink.” The long- and short-term drinkers and Control I group were used as the training sample, while the medium-term and Control II group were used as the test sample.

We used the European School Survey Project on Alcohol and Drugs [ESPAD (G. Schumann et al., 2010; Whelan et al., 2014)] for ages 14, 16, and 19 years to assess the alcohol consumption, smoking, and cannabis use. The ESPAD category scores are as follows: 0(0), 1(1–2), 2(3–5), 3(6–9), 4(10–19), 5(20–39), 6(≥40). The primary questions of interest concerned lifetime alcohol use [for example, On how many occasions (if any) in your lifetime have you had any alcoholic beverage?]; lifetime drunken episodes [for example, On how many occasions (if any) in your lifetime have you been drunk from drinking alcoholic beverages?]; lifetime smoking (for example, On how many occasions during your lifetime have you smoked cigarettes?); and lifetime cannabis use (for example, On how many occasions in your whole lifetime have you used marijuana or hashish?). Variables associated with the occasions of alcohol use and the episodes of binge drinking were measured at three time points (ages 14, 16, and 19), and the data were organized according to the duration and amount of binge drinking during adolescence (Fig. 1, eFig. 1). More details are provided in the eMethods.

#### 2.2.4 Cognition and personality

We used the Cambridge Neuropsychological Test Automated Battery [Cantab (Sahakian et al., 1988)], Monetary-Choice Questionnaire (KIRBY rate) (Kirby et al., 1999), personality characteristics tests, including the Revised NEO Personality Inventory [NEO-PI-R (Costa and Mccrae, 1992)], and the Substance Use Risk Profile Scale [SURPS (Woicik et al., 2009)] to assess cognition and personality factors. Further details are provided in the eMethods.

### 2.3 Statistical analyses

#### 2.3.1 Univariate approach

We used two-sample Student's *t*-tests, controlling for covariates (sex and site) for univariate analyses, and employed a false discovery rate (FDR) correction for multiple comparisons.

#### 2.3.2 Multivariate approach with hypothesis-guided feature selection

To identify discriminative features in high dimensional space and control for the over-fitting problem, we combined several dimension reduction approaches with the multivariate pattern analysis approach [Support-Vector Machine (SVM), (Suykens and Vandewalle, 1999)]. The main steps are described below and additional details are provided in the eMethods.

##### 2.3.2.1 Candidate feature selection and dimension reduction

**Fig. 2:**
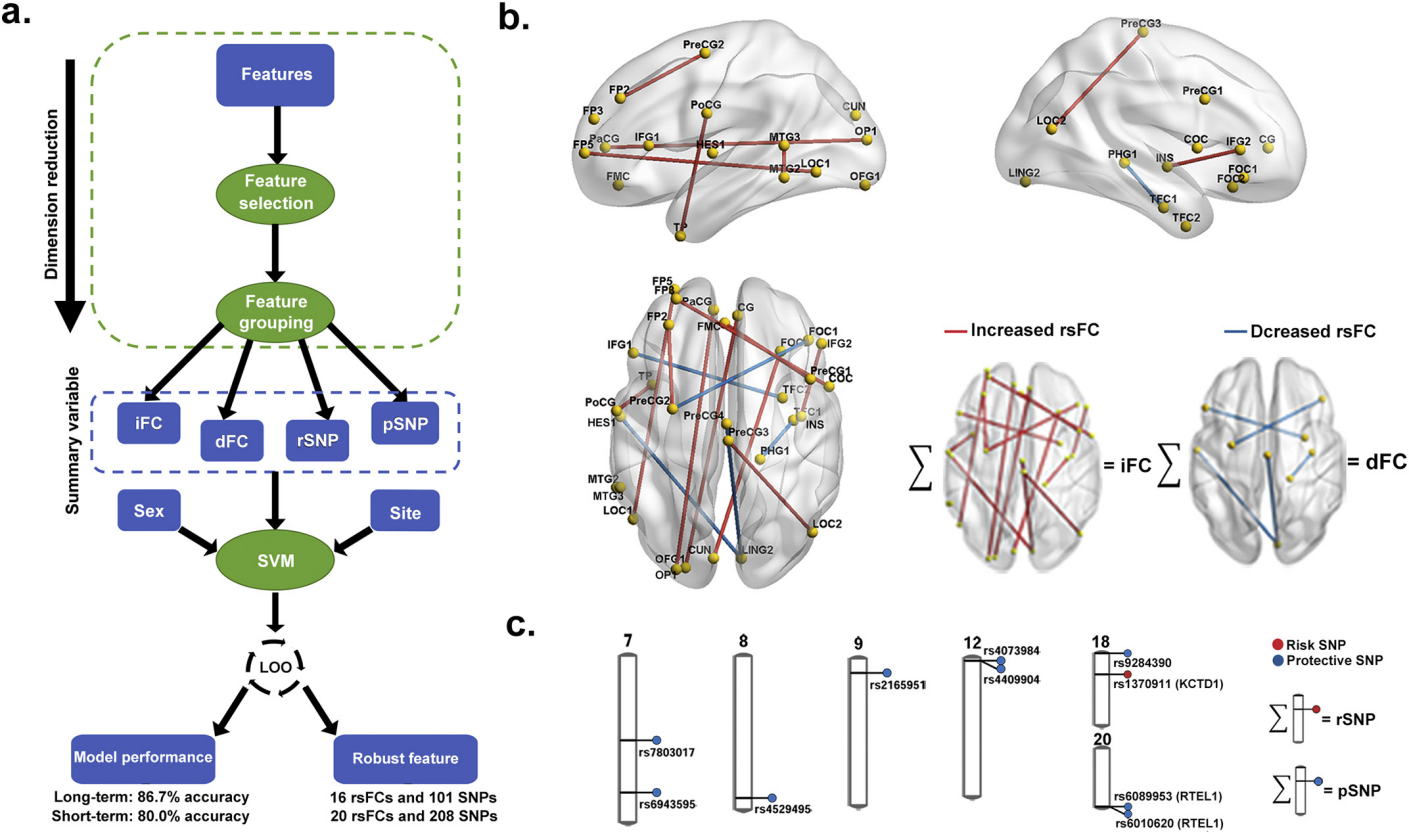
Discriminative brain changes and genetic markers for adolescent binge drinking. (A) Flowchart of model building to classify binge drinkers from non-binge controls in the training sample. (B) rsFC regions robustly selected by the SVM-long for long-term drinkers during the LOO procedure. The summary iFC/dFC score was summed over selected FC regions for drinkers (red, increased; blue, decreased) using the LOO procedure. PaCG: Paracingulate Gyrus; LOC: Lateral Occipital Cortex; PreCG: Precentral Gyrus; CG: Cingulate Gyrus; TP: Temporal Pole; PHG: Parahippocampal Gyrus; FMC: Fontal Medial Cortex; FOC: Fontal Orbital Cortex; CUN: Cuneal Cortex; TFC: Temporal Fusiform Cortex; PoCG: Postcentral Gyrus; MTG: Middle Temporal Gyrus; COC: Central Opercular Cortex; FP: Frontal Pole; OP: Occipital Pole; INS: Insular Cortex; IFG: Inferior Frontal Gyrus; HES: Heschl's Gyrus; OFG: Occipital Fusiform Gyrus; LING: Lingual Gyrus. (C) SNPs robustly selected by both SVM-long and SVM-short for long- and short-term drinkers during the LOO procedure. Summary rSNP/pSNP scores were summed over the selected SNPs for drinkers (red, risk; blue, protective) using the LOO procedure. Official gene symbols are displayed in brackets if the SNP is in a coding region. KCTD1: potassium channel tetramerization domain containing 1; RTEL1: regulator of telomere elongation helicase 1.

To reduce dimensionality, we first used the lasso-regularized logistic regression to select the most discriminative rsFCs between the binge drinkers and non-drinking controls. Chi-squared tests were used to select the SNPs with significantly different frequencies in subjects with a history of binge drinking compared to those without a binge drinking history. Next, instead of using these features as inputs to the SVM, we further reduced the dimensionality by calculating four summary scores, including increased/decreased rsFC (iFC/dFC) or risk/protective SNP (rSNP/pSNP) scores (Fig. 2A). To prevent model over-fitting in the training sample, we applied the leave-one-out (LOO) cross-validation procedure to the SVM, which was built by the two steps described above. Only those features (rsFC and SNP) that were repeatedly selected in >90% of iterations during the LOO were considered robustly discriminating features (namely, the candidate features) between the binge drinkers and the control subjects. We trained one SVM for the long-term drinkers (SVM-long classifier) and the other SVM for the short-term drinkers (SVM-short classifier). To assess the contribution of each domain (SNP, rsFC, and covariates) in classification, we compared the model performance before and after the removal of each domain from the model inputs. The candidate features in the domains significantly contributed to SVM-long and SVM-short were summarized into four new scores (iFC, dFC, rSNP, and pSNP) for next stage.

##### 2.3.2.2 Hypothesis-guided feature selection

**Fig. 3:**
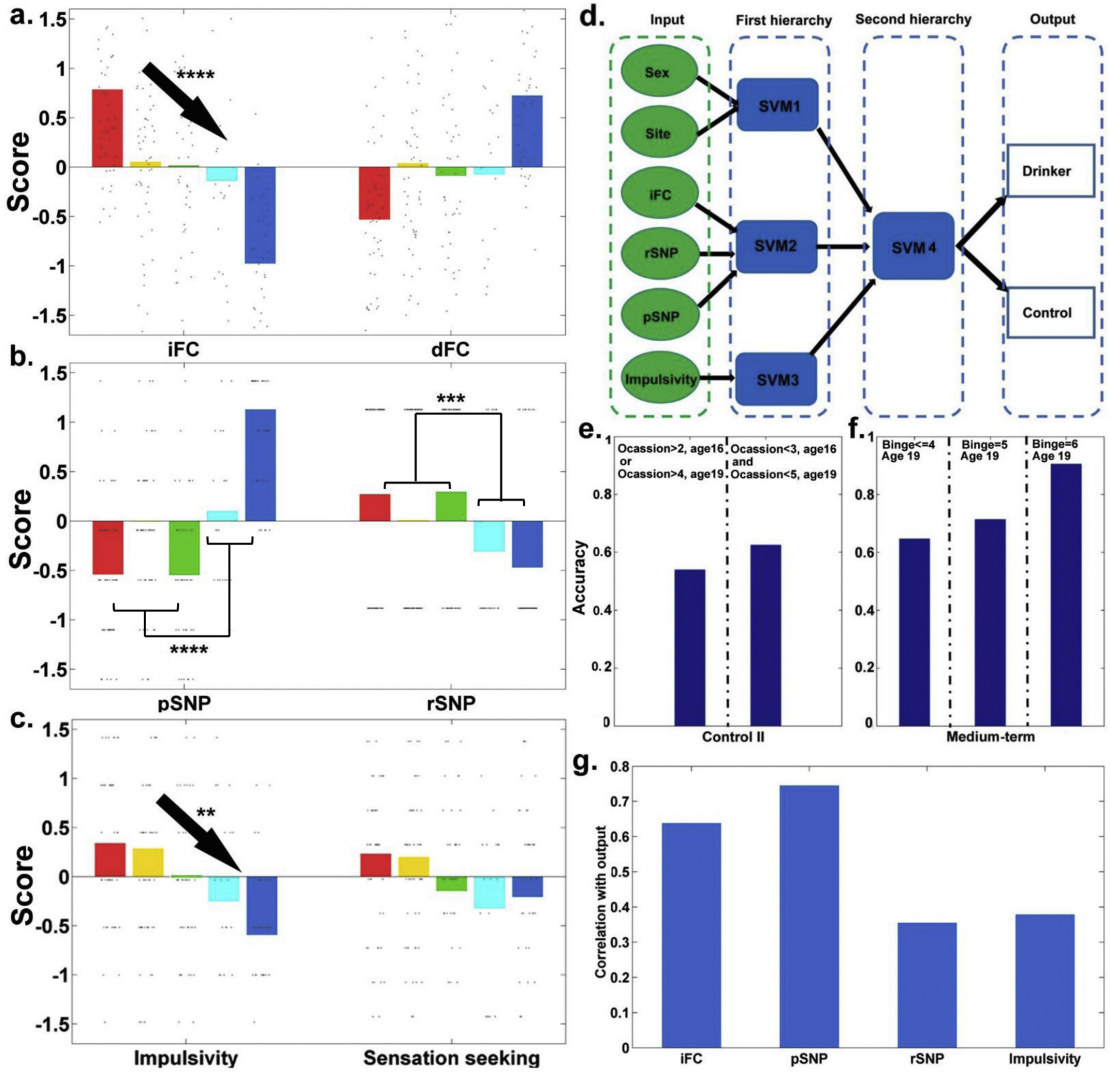
Selected features and hierarchical model. (A), (B), (C) Comparison of feature scores across the five groups. The black arrow indicates a significant monotonically decreasing trend confirmed by the Jonckheere-Terpstra trend test (****p < 10^−16^; ***p < 10^−4^; **p < 10^−2^). Five bars (groups) from left (red) to right (blue) were long-term, medium-term, short-term drinkers, Control II and Control I, respectively. (D) Structure of the hierarchical model for adolescent binge drinking. (E) Comparison of the classification accuracies using the SVM4 on the subgroups of Control II (test sample). This was stratified by lifetime drinking occasions, where fewer drinking occasions achieved higher accuracy. (F) Comparison of classification accuracies using the SVM4 on the subgroups of medium-term drinkers. This was stratified according to lifetime binge drinking by age 19, where a higher number of episodes of binge drinking achieved a higher accuracy. (G) Contribution of iFC, pSNP, rSNP, and impulsivity scores. Contribution was characterized by the absolute value of the correlation between the scores and the model output.

According to our hypothesis of trend for the consequences of adolescent binge drinking, we focused on the personality scores and the four new scores established by candidate features. We employed the Jonckheere-Terpstra trend test to identify the features that were consistent with the hypothesis of trend (i.e. the discriminative features having the largest, intermediate and smallest deviations from the control group in the binge drinkers with the longest, medium, and shortest history of binge drinking, respectively; Fig. 3A, B, and C).

##### 2.3.2.3 Hierarchical classifier

Finally, we built a hierarchical classifier, which consisted of two layers: the in-layer and the out-layer (Fig. 3D). To further reduce dimensionality, we constructed the in-layer with three SVMs taking the inputs from covariates (SVM1), rsFC and SNP (SVM2), and personality (SVM3). Each of these SVMs was trained using the LOO procedure on the training data, including the Control I group, the long-term binge drinking group, and the short-term binge drinking group. In the out-layer, one SVM (SVM4) received only 3 input values from the output values of SVM1–3 (a greater output value means more likely to be a binge drinker). With this hierarchical classifier, we significantly reduced the model complexity by taking the inputs from three kinds of features, so as to achieve a better generalizability. The model was then tested using an independent test sample (i.e. the medium-term drinkers and the Control II group).

#### 2.3.3 Longitudinal analysis

We could not conduct a longitudinal analysis on the rsfMRI data because we only had 32 subjects (18 binge drinkers and 14 controls) with rsfMRI data available at the age of 14 after participant selection (eFig. 1). However, personalities were assessed at three time points (ages 14, 16, and 19); therefore, we estimated changes in personality between 14 and 16 years (difference_16–14_) and between 16 and 19 years (difference_19–16_). To compare the developmental trajectory of personality between the two different time periods (i.e. from 14 to 16 and from 16 to 19), difference_16–14_ and difference_19–16_ in binge drinkers must be established before and after binge drinking onset, respectively, thus only the short-term drinkers satisfied this condition. These differences were compared before and after the onset of binge drinking (significance was given by 1000 permutations of the paired median test). By conducting an Analysis of Variance (ANOVA) with repeated measures using SPSS (Released 2011. IBM SPSS Statistics for Windows, Version 20.0. Armonk, NY: IBM Corp), we were able to compare the longitudinal evolutions of both the impulsivity and the sensation-seeking scores between the short-term drinkers and the control subjects. The same approach was also applied to difference_16–14_ and difference_19–16_. The covariates (sex and data collection sites) were regressing out from the measurements before the group comparisons.

## 3 Results

### 3.1 Stratification of binge drinkers by the onset age of binge drinking

All participants were stratified into 5 groups, including long-term (*n* = 54), medium-term (*n* = 52), and short-term (*n* = 41) drinkers; Control I (*n* = 44) and Control II (*n* = 21). These were further divided into the training and test samples (Fig. 1, eFig. 1 and Table 1). To summarize the analyses in this study, a diagram of the study design depicting the relationship between different analyses is presented in eFig. 2.

**Table 1:** Characteristics of the participants.

	Training sample			Independent sample	
	Control I	Long-term	Short-term	Control II	Medium-term
Group size	44	54	41	21	52
Sex (% female)	56.8%	57.4%	39.0%	81.0%	38.5%
Binge 14	0(0)	2.89(1.09)	0.02(0.16)	0(0)	0.06(0.24)
Binge 16	0(0)	3.92(1.71)	0.17(0.39)	0(0)	2.94(1.26)
Binge 19	0(0)	5.06(1.38)	3.56(1.25)	0(0)	4.88(1.20)
Cannabis use 14	0(0)	0.81(1.61)	0(0)	0(0)	0.039(0.19)
Cannabis use 16	0(0)	2.35(2.16)	0.17(0.95)	0(0)	1.5(2.06)
Cannabis use 19	0.11(0.54)	3.13(2.29)	0.39(0.80)	0(0)	2.89(2.34)
Smoking 14	0.16(0.75)	2.74(2.37)	0(0)	0.05(0.22)	0.21(0.70)
Smoking 16	0.27(1.17)	3.81(2.35)	0.02(0.15)	0.30(1.34)	2.75(2.64)
Smoking 19	0.16(0.78)^a^	4.50(1.89)	1.10(1.62)	0.50(1.40)	3.92(2.31)

Cognition					
Cantab 1	526.52(91.88)	505.64(94.89)	509.69(90.29)	525.05(112.16)	529.59(85.11)
Cantab 2	512.05(91.32)	485.20(82.78)	500.38(65.42)	485.14(98.47)	519.24(83.57)
Cantab 3	6.29(3.91)	6.28(5.10)	5.41(5.89)	5.69(2.95)	6.69(5.27)
Cantab 4	9.36(4.90)	7.62(5.18)	7.50(5.74)	6.69(3.64)	8.23(5.10)
Cantab 5	0.19(0.14)	0.19(0.11)	0.17(0.11)	0.16(0.11)	0.20(0.14)
Cantab 6	1715.03(436.70)	1656.80(492.85)	1385.54(283.19)	1504.74(455.70)	1612.39(502.22)
Cantab 7	0.46(0.12)	0.49(0.10)	0.49(0.09)	0.46(0.11)	0.51(0.12)
Cantab 8	0.95(0.06)	0.95(0.08)	0.98(0.04)	0.99(0.03)	0.95(0.07)
Cantab 9	2.09(0.89)	1.94(0.94)	1.84(0.67)	2.43(1.02)	1.99(1.03)
Cantab 10	0.51(0.13)	0.54(0.10)	0.54(0.10)	0.51(0.12)	0.56(0.13)
Cantab 11	97.44(7.37)	96.81(5.43)	96.01(6.09)	95.37(7.35)	93.56(10.26)
Cantab 12	0.93(0.04)	0.92(0.05)	0.93(0.05)	0.91(0.05)	0.94(0.04)
Cantab 13	10.53(11.71)	13.05(9.35)	11.54(13.44)	10.82(12.46)	10.16(9.38)
Cantab 14	27.31(6.38)	28.49(5.42)	27.00(6.13)	28.53(6.16)	27.47(5.68)
(K) Overall	0.01(0.02)	0.03(0.04)	0.02(0.02)	0.01(0.01)	0.02(0.03)
(K) Small	0.02(0.03)	0.05(0.05)	0.03(0.03)	0.03(0.04)	0.03(0.04)
(K) Medium	0.01(0.02)	0.03(0.04)	0.02(0.03)	0.01(0.02)	0.02(0.03)
(K) Large	0.01(0.01)	0.03(0.05)	0.02(0.03)	0.01(0.00)	0.01(0.02)
(K) Mean	0.01(0.02)	0.03(0.04)	0.02(0.02)	0.01(0.01)	0.02(0.02)

Personality					
(N) Neuroticism	17.82(7.99)	21.28(8.56)	19.68(6.58)	20.05(9.05)	21.82(8.96)
(N) Extraversion	26.75(6.35)	29.70(6.14)	30.07(5.61)	29.00(6.60)	30.52(6.09)
(N) Openness	28.80(4.83)	29.40(6.90)	26.46(5.66)	28.52(5.57)	29.00(6.04)
(N) Conscientiousness	31.55(6.23)	29.48(6.05)	32.37(4.92)	31.86(3.76)	30.92(5.62)
(N) Agreeableness	33.16(7.79)	29.04(6.56)	29.12(5.26)	33.57(3.67)	28.16(6.61)
(S) Anxiety	11.02(2.20)	11.82(2.93)	11.90(2.27)	12.35(2.78)	12.00(2.38)
(S) Negative Thinking	12.68(3.34)	12.73(3.49)	12.90(2.77)	12.50(3.66)	13.00(3.56)
(S) Impulsivity	9.84(1.75)	11.78(2.08)	11.07(1.85)	10.55(2.06)	11.66(2.02)
(S) Sensation Seeking	13.48(3.07)	14.73(2.84)	13.65(2.72)	13.15(3.08)	14.64(2.51)

Substance using behavior (lifetime binge drinking, cannabis use, and smoking) was assessed by the ESPAD at ages 14, 16, and 19, with the values representing the occasions of lifetime drunken episodes, lifetime cannabis use, and lifetime smoking, respectively.

Cantab, Cambridge Neuropsychological Test Automated Battery. Cantab 1–14: Affective Go-NoGo Latency Negative, Affective Go-NoGo Latency Positive, Affective Go-NoGo Omission Negative, Affective Go-NoGo Omission Positive, Delay Aversion, Deliberation Time, Overall Proportion Bet, Quality of Decision Making, Risk Adjustment, Risk Taking, Pattern Recognition Memory, Rapid Visual Processing, Spatial Working Memory of Errors, Spatial Working Memory of Strategy.

(K) represents the Monetary-Choice Questionnaire (KIRBY rate).

(N) represents NEO-PI-R, Revised NEO Personality Inventory.

(S) represents SURPS, Substance Use Risk Profile Scale.

More details about cognitive tests and personality questionnaires are provided in the eMethods.

Mean scores ± standard deviation are listed.

a One participant in the control group reported less lifetime smoking at age 19 than previously reported in the ages 14 and 16 years. One participant in the Control II group was missing data for lifetime smoking.

### 3.2 Discriminative features of binge drinkers

There were higher personality scores, including sensation seeking (*t*_84_ = 2.77, *p* = .007) and impulsivity (*t*_84_ = 4.76, *p* = 8.10 × 10^−6^) in the long-term binge drinkers than the Control I group at age 19, after FDR correction by univariate comparison. However, there were no significant differences in other personality or cognitive scores between controls and binge drinkers (eTable 1). We found no significant differences in rsFC or genetic variants between binge drinkers and not-binge controls (eFig. 3).

By constructing accurate multivariate classifiers [classification accuracy = 86.7%; area under curve (AUC) = 0.900, eFig. 4] for long-term drinkers that included sex and site of data collection as covariates (classifier SVM-long, Fig. 2A), we identified robust rsFC and SNP features (eTables 2, 3, 4a; Fig. 2C) in the long-term drinkers. Similarly, we identified robust features (eTables 4b-c) in the short-term drinkers (classifier SVM-short, classification accuracy = 80.0%, Fig. 2A; AUC = 0.846, eFig. 4b). The net reclassification improvement (NRI) test (Pencina et al., 2008) showed that the contribution of rsFC (eFig. 4c) was significant to the SVM-long classifier (NRI_95_ = 2.01, *p* = .044) but not the SVM-short (NRI_85_ = 0.22 and *p* = .825). In contrast, the SNPs significantly contributed to both classifiers (SVM-long: NRI_95_ = 2.90, *p* = .0038; SVM-short: NRI_85_ = 2.63, *p* = .0085). Thus, we summarized the candidate rsFCs selected by SVM-long into iFC and dFC scores (Fig. 2B), and summarized the candidate SNPs selected by both SVM-long and SVM-short into rSNP and pSNP scores (Fig. 2C).

Among the established summary scores (Fig. 3), we found that iFC (z_212_ = 8.54, *p* = 1.34 × 10^−17^) and impulsivity (z_212_ = 2.59, *p* = .0047) scores showed a trend that increased across controls, short-, medium-, and long-term binge drinkers (eFig. 5 and eTable 5). We also found higher rSNP scores (*t*_210_ = 4.20, *p* = 3.88 × 10^−5^) and lower pSNP scores (*t*_210_ = −9.09, *p* = 7.40 × 10^−17^) in binge drinkers than controls. Thus, the final classifier (SVM4) for binge drinkers was created from the iFC, impulsivity, and p/rSNP scores (Fig. 3D).

### 3.3 Validation of models using an independent sample

In the test sample, SVM4 achieved a classification accuracy of 71.2% for medium-term binge drinkers. The pSNP score contributed the most to this classifier (Fig. 3G; *r* = 0.75, *p* = 6.69 × 10^−26^), followed by the iFC score (*r* = 0.64, *p* = 2.88 × 10^−17^). The rSNP and the impulsivity scores showed relatively small, but significant, contributions (rSNP: *r* = 0.36, *p* = 1.79 × 10^−5^; impulsivity: *r* = 0.38, *p* = 6.94 × 10^−6^). The covariates, including sex and site of data collection, contributed to the classifier significantly less than the rsFC and SNP (0.235–0.539, 95% confidence interval of contribution difference by 5000 bootstraps, eFig. 6). Without the hierarchical design (eTable 6) or hypothesis-guided feature selection (eFig. 7), the multivariate model tended to over-fit the training sample, which significantly reduced the classification accuracy of the test sample. As the onset time of binge drinking may be different from the onset time of other substance use, the hypothesis-guided feature selection may improve the specificity of the selected features to be associated with binge drinking instead of general substance use. Indeed, applying the established classifier to identify cannabis users and smokers in the test sample yielded accuracies of 49.3% and 54.8%, respectively, which is comparable to chance.

In the Control II group, the accuracies decreased from 62.5% to 53.9% in subjects with increased Occasion scores (Fig. 3E). In the medium-term drinkers, classification accuracies were 64.7%, 71.4%, and 90.5% in subgroups of binge scores that were <4 (*n* = 17), equal to 5 (*n* = 14), or 6 (*n* = 21), respectively (Fig. 3F).

### 3.4 Onset of binge drinking slowed down the developmental trajectory of improvement in impulsivity

**Fig. 4:**
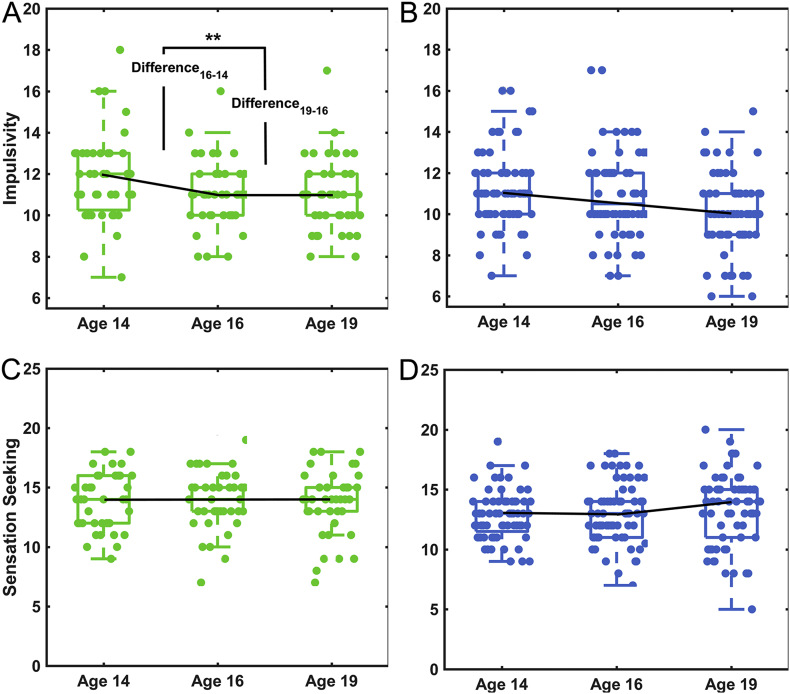
Analysis of the relationship between personality scores and binge drinking. (A), (B) Boxplots of “impulsivity” scores in the controls (blue) and the short-term drinkers (green) at different ages. Controls included Control I and Control II groups, as they did not binge drink at all. The statistical significance displayed in (A) refers to the statistical significance of the difference between difference_16–14_ of “impulsivity” scores and difference_19–16_ of “impulsivity” scores in short-term drinkers. (C), (D) Boxplots of “sensation seeking” scores in the controls (blue) and short-term drinkers (green) at different ages. Boxplots showing the median, 25th, and 75th percentiles; whiskers show ±2.7 standard deviation. (*p < 10^−1^, **p < 10^−2^, ***p < 10^−4^).

It was difficult to elucidate the cause and effect of binge drinking because the impulsivity score was higher in the binge drinkers (including the medium-term and the short-term groups) than controls (including Control I and Control II) at both ages (14: *t*_146_ = 2.14, *p* = .034; 19: *t*_145_ = 4.1, *p* = 6.93 × 10^−5^). For each period (14–16, or 16–19), we computed the difference between two time points to represent the developmental improvements in impulsivity. In controls (including Control I and Control II), we observed that impulsivity improved at a steady speed from ages 14 to 16 and 16 to 19 (Fig. 4B). However, in short-term drinkers (*n* = 41), we found that the improvement in impulsivity between ages 16 and 19 was lower than between ages 14 and 16 (*p* = .003, Fig. 4A). This effect was not observed for sensation seeking (Fig. 4C–D). Using ANOVA with repeated measures, we identified an interaction effect between time (ages 14, 16 and 19) and group (short-term drinkers and controls) on impulsivity (*F*_97_ = 3.745, *p* = .027) and the developmental change of impulsivity (i.e. difference_16–14_ and difference_19–16_) (*F*_97_ = 5.516, p = .021). This interaction effect was not observed for sensation seeking.

## 4 Discussion

We conducted a systematical study to delineate the consequences of binge drinking in multiple domains, including the brain, personality, and cognition of adolescents, while considering multiple confounders, including genetics and other substance use. In the brain, we found that the frontal connectivity significantly contributed to the binge-drinking associated brain feature (11 out of 16 links selected for the iFC score). In the domain of personality, the developmental improvement of impulsivity was slowed down after the onset of binge drinking during adolescence. The current findings identified the disruptive effects of adolescent binge drinking on the developmental trajectories of both brain and personality.

The functional connectivity between the frontal cortex and precentral gyrus and occipital cortex are particularly vulnerable to adolescent binge drinking. The standard developmental trajectory of the functional architecture is to segregate the brain into different functional systems; for example, interactions among frontal and sensory-motor regions decrease with age (Gu et al., 2015; Stevens et al., 2009); and frontal areas undergo significant pruning processes during adolescence (Gogtay et al., 2004). It has been reported that the earlier age of the first drink of alcohol predicted the poorer performances in both the psychomotor speed and the visual attention (Nguyen-Louie et al., 2017), and the earlier onset age of the weekly drinking was associated with higher frontoparietal context-dependent functional connectivity between the bilateral posterior cingulate and both cortical and subcortical areas implicated in the attentional processes in young adults (Nguyen-Louie et al., 2018). Our results indicated an accumulating effect of adolescent binge drinking on frontal connectivity because more episodes of binge drinking increased the accuracy of the classification between binge drinkers and non-binge controls. Therefore, if binge drinking is not totally avoidable in adolescents, efforts should be considered to control the number of incidences.

The maturation process of personality is characterized by a decline in negative emotionality (Blonigen et al., 2008; Donnellan et al., 2007); however, we found that the standard decline of impulsivity was slowed down by the onset of adolescent binge drinking. Compared with our observation of sensation seeking, it suggests that the adolescent binge drinking may be particularly disruptive to the developmental processes normally taking place at the time period that is later than the onset age of binge drinking. Interestingly, sensation seeking becomes relatively stable after mid-adolescence, while impulsivity shows an explicitly monotonic decline through adolescence (Harden and Tucker-Drob, 2011; Steinberg et al., 2008). Therefore, adolescent binge drinking had a greater impact on the maturation of impulsivity but not sensation seeking. Furthermore, we found higher impulsivity scores in the binge drinkers than in controls at baseline (before the onset of binge drinking), which is consistent with previous studies that used the impulsivity score as a predictor for later alcohol misuse (Chassin et al., 2004; Elkins et al., 2006; King and Chassin, 2004). Therefore, the current findings provide longitudinal evidence to suggest that there is a negative feedback loop between impulsivity and adolescent drinking behavior.

Increased functional connectivity and impulsivity scores both contributed to our classification model for binge drinkers independent of genetic features; therefore, these features are unlikely to be independent manifestations of common genetic predispositions for adolescent binge drinking. In fact, gene expression patterns in the brain can be altered by alcohol intake, especially in the frontal areas (Lewohl et al., 2000; Liu et al., 2006; Mayfield et al., 2002). This altered expression pattern may lead to dysfunctional brain connectivity; rsFC has been correlated with gene expression linked to ion channel activity and synaptic function (Richiardi et al., 2015). In addition, the brain serotonin system is responsible for impulsivity (Leyton et al., 2001; Sachs and Dodson, 2017) and this system can be disturbed by alcohol (Burnett et al., 2012; Sachs and Dodson, 2017; Shibasaki et al., 2010), which may account for the impact of binge drinking on the developmental trajectory of impulsivity in this study.

The advantages of this study include the longitudinal design, independent test sample, and disassociation with cannabis use. However, several limitations must be considered when interpreting our results. First, we used binge drinking as a behavioral indicator for alcohol-induced neurotoxicity, which is not necessarily comparable to clinically diagnosed alcohol dependency. We measured drinking behavior at ages 14, 16, and 19 only; therefore, more detailed measurements outlining the frequency and quantity of drinking would assist with the identification of heavy drinkers from occasional drinkers. Second, a limited number of individuals in the IMAGEN study participated in the rsfMRI experiments at age 14, and only 24 were defined as binge drinkers across the three binge drinking groups. Future longitudinal studies employing rsfMRI at baseline will enable estimations of developmental trajectories of rsFC before and after the onset of binge drinking and thereby, provide more insights into the disruptive effects of adolescent binge drinking on the developmental trajectory of brain functional architecture.

## 5 Conclusions

We found new evidence for disrupted brain functional organization in adolescents who participate in binge drinking behaviors and highlighted a negative feedback loop that interacted with impulsivity following binge drinking during early adolescence. Alcohol is the most abused substance in adolescents; therefore, its effect on the brain and personality must be considered in remedy programs to prevent further development of alcohol-related adverse outcomes. The identified disruptive effects of adolescent binge drinking provide potential targets for such interventions in adolescents with a history of binge drinking.

## Data availability

IMAGEN data are available by application to consortium coordinator Dr. G. Schumann (http://imagen-europe.com) after evaluation according to an established procedure.

## Code availability

Matlab codes for main algorithms used in this work are provided at the following website https://github.com/qluo2018/RetraceHistoryOfBingeDrinking.

## Funding information

Dr. Luo was supported by National Key Research and Development Program of China (grant 2018YFC0910503), the National Natural Science Foundation of China (grants 81873909), the Natural Science Foundation of Shanghai (grant 17ZR1444400), Shanghai Municipal Science and Technology Major Project (grant 2018SHZDZX01), and Zhangjiang Lab. Dr. Feng was partially supported by the key project of Shanghai Science and Technology Innovation Plan (grant 16JC1420402), the National Natural Science Foundation of China (grant 91630314), the Shanghai AI Platform for Diagnosis and Treatment of Brain Diseases, the Project of Zhangjiang Hi-Tech District Management Committee, Shanghai (grant 2016-17) and the 111 Project (grant B18015). Dr. Feng was a Royal Society Wolfson Research Merit Award holder. Dr. Li was partially supported by the Shanghai Municipal Commission of Health and Family Planning (grants 2017ZZ02026, 2018BR33, 2017EKHWYX-02 and GDEK201709), Shanghai Shenkang Hospital Development Center (grant 16CR2025B), Shanghai Municipal Education Commission (grant 20152234), National Natural Science Foundation of China (grants 81571031, 81761128035, and 81703249), Shanghai Committee of Science and Technology (grants 17XD1403200 and 18DZ2313505), Xinhua Hospital of Shanghai Jiao Tong University School of Medicine (grants 2018YJRC03, talent introduction-014, and Top talent-201603). This work also received support from the following sources: the European Union-funded FP6 Integrated Project IMAGEN (Reinforcement-related behavior in normal brain function and psychopathology) (LSHM-CT- 2007-037286), the Horizon 2020 funded ERC Advanced Grant “STRATIFY” (Brain network based stratification of reinforcement-related disorders) (695313), ERANID (Understanding the Interplay between Cultural, Biological and Subjective Factors in Drug Use Pathways) (PR-ST-0416-10004), BRIDGET (JPND: BRain Imaging, cognition Dementia and next generation GEnomics) (MR/N027558/1), the FP7 projects IMAGEMEND (602450; IMAging GEnetics for MENtal Disorders) and MATRICS (603016), the Innovative Medicine Initiative Project EU-AIMS (115300-2), the Medical Research Council Grant “c-VEDA” (Consortium on Vulnerability to Externalizing Disorders and Addictions) (MR/N000390/1), the Swedish Research Council FORMAS, the Medical Research Council, the National Institute for Health Research (NIHR) Biomedical Research Centre at South London and Maudsley NHS Foundation Trust and King's College London, the Bundesministeriumfür Bildung und Forschung (BMBF grants 01GS08152; 01EV0711; eMED SysAlc01ZX1311A; Forschungsnetz AERIAL 01EE1406A), the Deutsche Forschungsgemeinschaft (DFG grants SM 80/7-1, SM 80/7-2, SFB 940/1). Further support was provided by grants from: ANR (project AF12-NEUR0008-01-WM2NA, and ANR-12-SAMA-0004), the Fondation de France, the Fondation pour la Recherche Médicale, the Mission Interministérielle de Lutte-contre-les-Drogues-et-les-Conduites-Addictives (MILDECA), the Assistance-Publique-Hôpitaux-de-Paris and INSERM (interface grant), Paris Sud University IDEX 2012; the National Institutes of Health, Science Foundation Ireland (16/ERCD/3797), U.S.A. (Axon, Testosterone and Mental Health during Adolescence; RO1 MH085772-01A1), and by NIH Consortium grant U54 EB020403, supported by a cross-NIH alliance that funds Big Data to Knowledge Centres of Excellence.

## Disclosures

Dr. Banaschewski has served as an advisor or consultant to Actelion, Hexal Pharma, Lilly, Lundbeck, Medice, Neurim Pharmaceuticals, Novartis, Shire. He received conference support or speaker's fee by Lilly, Medice, Novartis and Shire. He has been involved in clinical trials conducted by Shire & Viforpharma; the present work is unrelated to these relationships. Dr. Walter received a speaker honorarium from Servier (2014). All other authors declare no conflict of interest. The other authors report no biomedical financial interests or potential conflicts of interest.

## Contributions

Q.L. and J.F., G.S. conceived the project. H.R., Y.Z. and Q.L. performed most of the analyses. G.R., S.D., E.B. and F.L. analysed behavioral data. Z.L., H.R., T.B., A.B., U.B., C.B., H.F., V.F., H.G., P.G., A.H., B.I., J.M., M.P., F.N., D.O., L.P., S.H., J.F., M.S. H.W., and R.W. acquired and processed neuroimaging, genetic and behaviour data. Q.L., Y.Z. and H.R. wrote the manuscript. G.S. and J.F. edited the manuscript. All authors reviewed the manuscript and discussed the work.

## Appendix A Supplementary data

Supplementary material Image 1
