# Supplementary material for: Adolescent binge drinking disrupts normal trajectories of brain functional organization and personality maturation
Source: Neuroimage Clin. 2019 Mar 31;22:101804. doi: 10.1016/j.nicl.2019.101804 (PMC6451196; doi:10.1016/j.nicl.2019.101804)
Supplement: Supplementary file 1 — Supplementary material [file mmc1.docx]

**Supplementary information:**

**Adolescent binge drinking disrupts normal trajectories of brain functional organization and personality maturation**

Hongtao Ruan, et al.

Table of Contents

[Data acquisition and preprocessing 3](#_Toc2952578)

[Cognitive tests and personality questionnaires 6](#_Toc2952579)

[Stratified groups and demographic characteristics 8](#_Toc2952580)

[Hypothesis-guided integration of multi-scale data for classification (HIMuDC) 10](#_Toc2952581)

[Parameter determination for SVMs 14](#_Toc2952582)

[Excluding potential confounding effects 16](#_Toc2952583)

[eFigure 1. Selected participants and stratified groups 17](#_Toc2952584)

[eFigure 2. Overview of the study design 18](#_Toc2952585)

[eFigure 3. Manhattan plot of rsFCs and SNPs 19](#_Toc2952586)

[eFigure 4. Performances of different classifiers for binge drinkers and contributions of different features to these models 20](#_Toc2952587)

[eFigure 5. Comparison on increased rsFCs 21](#_Toc2952588)

[eFigure 6. Comparison of the contributions by the *SVM*1, *SVM*2, and *SVM*3 to the final model for binge drinking 22](#_Toc2952589)

[eFigure 7. ROC curves of the *SVM*4 and SVM-comparisons 23](#_Toc2952590)

[eTable 1. Univariate comparison on cognition tests and personality questionnaires 24](#_Toc2952591)

[eTable 2. Correlation between the featured rsFC and the long-term binge drinking 25](#_Toc2952592)

[eTable 3. Abbreviations for brain regions in this study 26](#_Toc2952593)

[eTable 4a. Discriminative SNPs selected by the multivariate model for long-term drinkers 28](#_Toc2952594)

[eTable 4b. Discriminative FCs selected by the multivariate model for short-term drinkers 30](#_Toc2952595)

[eTable 4c. Discriminative SNPs selected by the multivariate model for short-term drinkers 31](#_Toc2952596)

[eTable 5. Summary statistics of the increased rsFCs in five groups 34](#_Toc2952597)

[eTable 6. Comparison of classification accuracies in each group by different models 35](#_Toc2952598)

[IMAGEN consortium author list 37](#_Toc2952599)

**Data acquisition and preprocessing**

***Resting-state functional imaging data preprocessing***

The functional magnetic resonance imaging data used in this multicenter study were obtained using 3T Siemens MRI scanners at seven study sites with a set of parameters compatible with all scanners, and both phantom and *in vivo* QC procedures were employed to ensure comparable image quality including signal-to-noise level across scanning sites ([Richiardi et al., 2015](#_ENREF_6); [Schumann et al., 2010](#_ENREF_8)). The first ten volumes were removed to ensure that magnetization equilibrium was reached. The functional images consist of a 6.5 min echo-planar imaging resting-state scan with TR/TE/Flip Angle = 2200 ms/30 ms/75°, 64 × 64 × 40 voxels with 2.4 mm slice thickness, 1 mm slice gap, and a field of view of 218 × 218 mm, yielding isotopic 3.4mm voxels. The structural image consists of a T1-weighted MPRAGE image of 256 × 256 × 160/166 voxels (dependent on the manufacturer), with a 1.1 mm isotropic voxel size. Other parameters were based on the ADNI protocol (see http://adni.loni.usc.edu/methods/documents/mri-protocols/); full details are provided in the IMAGEN paper ([Schumann et al., 2010](#_ENREF_8)). Participants were required to keep their eyes closed, relaxed, but not sleep, during the resting-state session. All imaging data were realigned and normalized (by T1 unified segmentation) to a standard template (Montreal Neurological Institute) and resampled to 3 × 3 × 3 mm. All fMRI time-series underwent band pass temporal filtering (0.01–0.08 Hz); nuisance signal removal from the ventricles, deep white matter, global mean signal, CSF signal; and Friston 24 rigid-body motion correction parameters. We carefully performed the following procedures to achieve motion correction: three-dimensional motion correction and “scrubbing” ([Power, Barnes, Snyder, Schlaggar, & Petersen, 2012](#_ENREF_5)). Frames with frame-wise displacement >0.5 mm were deleted together with one preceding and two succeeding frames. After this quality control, 815 individuals remained. For each individual, we calculated the resting-state functional connectivity (rsFC) between each pair of brain regions (19,900 links for 200 atlas-defined brain regions, each of which had been parcellated to have homogenous whole-brain rsFC by a voxel-wise clustering algorithm ([Craddock, James, Holtzheimer, Hu, & Mayberg, 2012](#_ENREF_2)). The atlas is available at <http://ccraddock.github.io/cluster_roi/atlases.html>. As recommended by the authors, we chose the atlas (CC200) generated by a two-level clustering scheme for 200 brain regions (this number of brain regions is optimal for our analysis as we need to reduce the dimensionality to a low number and also preserve functional homogeneity and interpretability), using temporal correlation between voxel time-courses (tcorr, which is more stable than the spatial correlation). eTable 3 lists the abbreviations of those brain regions mentioned in the current work, as well as their locations compared with Harvard-Oxford Cortical Structural Atlas. This atlas is also recommended by the ABIDE (Autism Brain Imaging Data Exchange, <http://preprocessed-connectomes-project.org/abide/Pipelines.html>).

***Genome-wide genotype data acquisition and preprocessing***

A total of 2,087 IMAGEN blood samples were genotyped using the Illumina (Little Chesterford, UK) Human610-Quad Beadchip (N = 705) or Illumina Human660- Quad Beadchip (N = 1,382). DNA purification and genotyping were performed by the Centre National de Génotypage in Paris. DNA was purified from whole-blood samples (~10 ml) preserved in BD V acutainer EDTA tubes (Becton, Dickinson and Company, Oxford, UK) using the Gentra Puregene Blood Kit (Qiagen, Manchester, UK) according to the manufacturer’s instructions. For each genotyping platform the following quality control was performed separately. Single-nucleotide polymorphisms (SNPs) with call rates <95%, minor allele frequency <5%, and deviation from the Hardy–Weinberg equilibrium (P <1 × 10^−3^) were excluded from the analyses. Individuals showing an over- or under-abundance of heterozygosity (>5 d.d from the mean) were excluded from the subsequence analysis. Finally, we obtained 324,959 SNPs without any missing values in 764 individuals. We converted all SNPs into a binary value, wherein 0 indicated no mutation on that SNP and 1 indicated a mutation was present.

**Cognitive tests and personality questionnaires**

***Cantab***

All detail information of the Cantab ([Sahakian et al., 1988](#_ENREF_7)) can be found on the website, [www.cambridgecognition.com](http://www.cambridgecognition.com). Specifically, we used 14 items: affective go-nogo latency negative, affective go-nogo latency positive, affective go-nogo omission negative, affective go-nogo omission positive, delay aversion, deliberation time, overall proportion bet, quality of decision making, risk adjustment, risk taking, pattern recognition memory, rapid visual processing, spatial working memory of errors, and spatial working memory of strategy.

***KIRBY***

Delay discounting refers to the phenomenon that rewords decreases in subjective value as the delay associated with their receipt increases. KIRBY ([Kirby, Petry, & Bickel, 1999](#_ENREF_3)) is a common measure to assess delay discounting. After the test, each participant was assigned a *k* value which represents the discount rate of this participant. In our study, we examined 5 types of *k*: overall, (geometric) mean, small, medium, and large.

***NEO***

The NEO is a concise measure of five major domains of personality ([Costa & Mccrae, 1992](#_ENREF_1)). In this study, we included data from all 5 domains: extraversion, agreeableness, conscientiousness, neuroticism, and openness to experience.

***SURPS***

The SURPS is a questionnaire measuring the risk of substance use ([Woicik, Stewart, Pihl, & Conrod, 2009](#_ENREF_11)). It hypothesizes that four personality dimensions differentially relate to specific patterns of substance use. In this work, we investigated all 4 personality dimensions: negative thinking, anxiety, impulsivity, and sensation seeking.

**Stratified groups and demographic characteristics**

Three binge-drinking groups, namely the long-term [a minimum of three lifetime drunk episodes by age 14 corresponding to a “Binge (ESPAD)” score of 2]; medium-term [a maximum of two drink occasions corresponding to an “Occasion (ESPAD)” score less than 2 by age 14 and a minimum of three lifetime binge drinking episodes by age 16]; and short-term drinkers (a maximum of two drink occasions by age 16 and a minimum of three lifetime drunk episodes by age 19), had onset ages of binge before age 14, between the ages of 14 and 16, and after the age of 16, respectively. Two non-bingeing groups, including control I [no lifetime binge by age 19 and a maximum of nine lifetime uses of alcohol corresponding to an “Occasion (ESPAD)” score less than 4 until at least age 19] and control II [no lifetime binge drinking episodes by age 19, a maximum of nine lifetime uses of alcohol corresponding to an “Occasion (ESPAD)” score less than 4 by age 16, a maximum of 39 lifetime uses of alcohol corresponding to an “Occasion (ESPAD)" score less than 6 by age 19, and excluding participants in control I] groups, had the fewest and second fewest occasions of alcoholic drinking during their lifetimes, respectively ([Whelan et al., 2014](#_ENREF_10)). We found no sex differences between groups, but significant differences (χ^2^ = 65.70, p = 9.62 × 10^–6^) in geographic distribution, with more drinkers living in London (21.8%) or Nottingham (21.8%) and more controls living in Paris (22.8%) or Mannheim (22.8%). Many drinkers were also users of other substances (Table 1). In the long-term drinkers, 29.6% (n = 16) had used cannabis and 77.8% had smoked at least once in their lifetime by age 14. In the medium-term drinkers, 42.3% (n = 22) and 46.6 (n = 19) were cannabis users and smokers, respectively, by age 16. We also found 26.8% (n = 11) and 46.3% (n = 19) of the short-term drinkers had a history of cannabis consumption and smoking by age 19, respectively. All three drinking groups are binge drinkers from the same population. The hypothesis is that they might have damages to different extent. By identifying groups of extreme drinkers with the longest and the shortest histories of binge drinking in our sample, we were able to select the features that were associated with binge drinking and that also satisfied the *hypothesis of trend*, and expected to see an intermediate disruptive effect of these features in a medium-term history of binge drinking group.

**Hypothesis-guided integration of multi-scale data for classification (HIMuDC)**

We built the SVM ([Suykens & Vandewalle, 1999](#_ENREF_9)) with linear kernel to trace the binge-drinking behavior to earlier ages during adolescence by using the rsFC at age 19 and/or the genomic information. We used the following strategies to reduce the dimensionality of the feature space for the SVM to control for the risk of overfitting.

***Stage 1: Identifying effective and robust features***

First, we selected the top 300 most discriminative rsFCs between the drinking and control groups. Second, we used lasso-regularized logistic regression to further reduce the dimensionality in the rsFC. Third, according to negative or positive correlation between rsFC and the binge-drinking behavior, the selected rsFCs were grouped into the decreased or increased group, and the rsFCs were added together resulting one summary score for each group, namely an increased rsFC (iFC) or decreased rsFC (dFC) score.

We identified risk SNPs for binge drinking by *Chi*-square test between the drinking and control groups. With a threshold of 4.4 × 10^–4^ and 1.0 × 10^–3^ for long-term and short-term drinkers, respectively, the significant risk SNPs were selected. We established a risk score of SNP (rSNP) and a protective score of SNP (pSNP) by summing the selected SNPs that were positively and negatively associated with the binge-drinking behavior.

Using the LOO procedure, we trained an SVM with two rsFC scores, two SNP scores, and covariates (sex and site where the imaging data was acquired) for long-term and short-term drinkers respectively, SVM-long and SVM-short*.* We evaluated overall model performance using the area under the curve (AUC) of the receiver-operator characteristic (ROC) curve. SNPs and rsFCs that were repeatedly selected in >90% of iterations during the LOO procedure were considered robustly discriminating features (namely candidate features) between the binge drinkers and the control subjects ([Whelan et al., 2014](#_ENREF_10)).

In our model, we included three domains: SNP, rsFC, and covariates. To assess the contribution of each domain to the model, we compared the model performance before and after the removal of each domain from the model inputs. The significance of these differences in model performance was tested using one-tailed net reclassification improvement (NRI), which is a statistical test to compare AUCs ([Pencina, Agostino, & Vasan, 2008](#_ENREF_4)). We utilized the SVM-long and SVM-short classifiers to ensure that the domains we selected for classification were effective and robust to identify binge drinkers. We found that the contribution of rsFC was significant to the SVM-long classifier but not the SVM-short, and that the SNPs significantly contributed to both classifiers. Thus we summarized the candidate rsFCs selected by SVM-long and the candidate SNPs selected by both SVM-long and SVM-short into four types of scores (iFC, dFC, rSNP, and pSNP) for next stage.

**Stage 2: *Selecting features that support the hypothesis***

The SVM-long and SVM-short established the long-term and short-term binge drinkers, respectively. Next, we associated measureable biological features, such as brain connectivity and genetic variants, with different onset ages of binge drinking. We found brain changes, which were the selected features of the rsFC, contributed uniquely to the accurate classification of binge drinkers from the non-binge controls in the *SVM-long*, but not *SVM-short* group. This is consistent to our hypothesis that long-term drinkers might have more detectable differences from the non-binge controls, compared with the short-term drinkers. Compared with non-binge controls, we statistically tested the descending order from the long-term, medium-term, and short-term drinkers in the iFC score established for the long-term drinkers by the Jonckheere-Terpstra trend test. The same test was performed for “impulsivity” and “sensation seeking” to detect a significant descending trend. Meanwhile, genetic predispositions contributed significantly to both the *SVM-long* and the *SVM-short* groups, suggesting that specific and common genetic variants are associated with different onset ages of bingeing in adolescents. Furthermore, common genetic variants might be discriminative between binge drinkers and controls in general. In addition, SNP risk score in the three drinker groups were all higher than the two control groups. We assessed this genetic association using *Chi*-square test.

***Stage 3: Building a model with increased generalizability***

We combined the long-term and short-term drinkers into one drinking group, in order to train our model to classify drinkers from controls. We built one model (*SVM*1) for covariates (sex and site). For genetic predisposition (SNP risk identified by *SVM-long* and *SVM-short*) and brain changes (positively relevant rsFC score established in the *SVM-long*), we created a different model (*SVM*2). In addition, we also created a model (*SVM3*) for “impulsivity.” *SVM*1, *SVM*2, and *SVM3* were constructed using a Gaussian kernel function. The kernel parameter, γ, for each SVM was determined using the LOO procedure on the training data. The parameter was set to correspond to the median of classification accuracies resulting from all candidate parameters. The numeric output values (positive output indicated a drinker and negative output indicated a control; the higher the absolute value of the output the more confidence for the participant with the inputting features to be classified by the SVM) instead of a category label of *SVM*1*, SVM*2, and *SVM*3 were used as the input of an out-layer SVM, namely *SVM*4 with a linear kernel function. We trained *SVM*4 on drinker group and controls and then tested it on the test sample (medium-term drinkers and Control II).

To compare the contributions of different features to the output of *SVM*4, we calculated the point-biserial correlation, which is a correlation statistic between binary and continuous variables, in the training sample between the binary output of *SVM*4 and each individual feature, including the rsFC, risk SNP, “impulsivity”, sex, and site.

**Parameter determination for SVMs**

***Parameter determination for building SVM-long and SVM-short***

The parameters for building the SVM-*long* and SVM-*short* were determined using the LOO procedure. We successively removed one individual as the test sample and trained our model on all other individuals (training sample).

Before training, rsFCs with smallest *p* values resulting from two sample two-tailed *t-*tests on the training sample were preselected. The threshold, *N,* was set to range from 100–400 in 100 increments to detect the best performance. Next, we performed a *lasso*-regularized logistic regression, with an input of *N* rsFCs, on the training sample. The regularization parameter$\lambda$was set to range from 1 × 10^–4^ to 4 × 10^–4^, in increments of 1 × 10^–5^. rsFCs with a non-zero beta resulting from the *lasso*-regularized logistic regression were selected to build the SVM.

The most likely relevant SNPs were identified using *Chi-*square tests in the training samples. All SNPs with a *p*-value resulting from *Chi-*square test smaller than threshold *P* were chosen to be the relevant SNPs. We set the threshold *P* to range from$4\times10^{-4}$to$1.5\times10^{-3}$with increment$2\times10^{-5}$.

The constant constraint, *C,* in the SVM training algorithm was set to range from 0.005–0.1, in increments of 0.005. The optimal parameter set [*N*,$\lambda$, *P*, *C*] was chosen from the highest prediction accuracy on the test sample, and was finally set as [300, 1.3 × 10^–4^, 1.3 × 10^–4^, 1.3 × 10^–4^, 4.4 × 10^–4^, 0.007],for building SVM-*long* and [300, 3.5 × 10^–4^, 1.0 × 10^–3^, 0.07] for building SVM-*short*.

***Parameter determination for building the hierarchical model***

In the hierarchical model, *SVM*1, *SVM2,* and *SVM*3 were all constructed with a Gaussian kernel function. Thus, we determined the kernel parameter$\gamma$for *SVM*1, *SVM2,* and *SVM*3 using the LOO procedure.

For *SVM*1, *SVM*2 and *SVM*3, similarly to the method described in last section, we successively took removed one individual as the test sample and trained an SVM on the training sample. The kernel parameter, γ, was set to range from 0.1 to 2, in increments of 0.1. The constant constraint, *C,* was set to use values from [0.001, 0.005, 0.0, 0.05, 0.1, 1.0, 1.5, 2.0, 2.5, 3.0]. In order to avoid overfitting, γ and *C,* were set to correspond to the median of the classification accuracies resulting from all candidate γ in the testing sample. Ultimately, γ was chosen to be 1.8, 0.8, and 1 for *SVM*1, *SVM*2, and *SVM*3 respectively.

The out-layer model, *SVM*4, was constructed using a linear kernel function, and the constant constraint, *C,* was at 0.1, which was determined using the same procedure.

**Excluding potential confounding effects**

***Classification of substance users***

Individuals in the long-term drinker and control I groups with at least 1 lifetime use of cigarettes by age 14 were considered as cigarette users. Those with zero use of cigarettes by age 14 were considered as non-users. Similarly, an individual with ≥1 or 0 lifetime uses of cannabis by age 14 was considered as a cannabis user or non-user, respectively. We applied the SVM-*long* to all long-term drinkers and controls in the control I group. If SVM-*long* returned a label of 1 or 0, then the corresponding subject was classified as a “user” or “non-user,” respectively. The accuracy of substance user classification was the amount of overlap between the labels provided by SVM-*long* and the true labels.

The same procedures were performed on the short-term drinker and control I groups, except the user or non-user status was determined by ≥1 use prior to age 19 instead of 14. The SVM-*short* was applied to these individuals to assess its accuracy.

Similarly, independent samples were determined as users and non-users according to if an individual had ≥1 lifetime use by age 16. The *SVM*4 was used to identify these labels and assessed for accuracy in the same way.

**
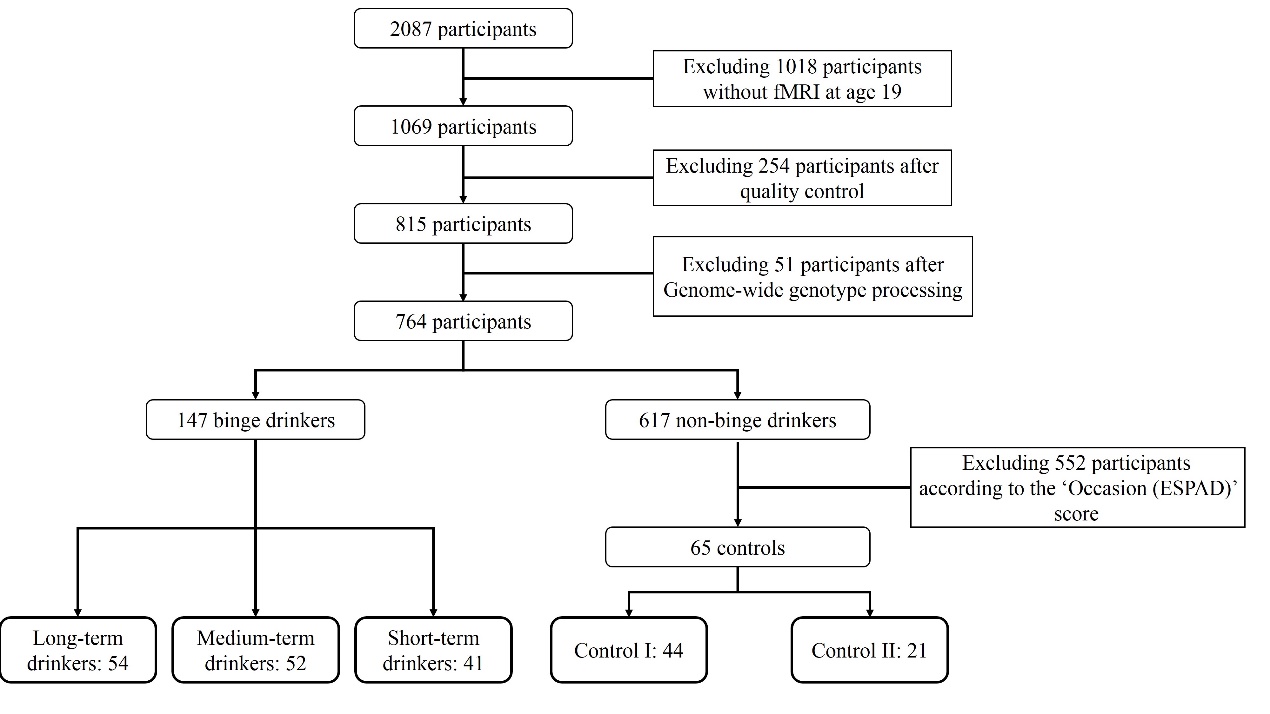
**

# **eFigure 1. Selected participants and stratified groups**

We selected our study sample according to the duration and amount of binge drinking during adolescence using the parameters: occasions of alcohol use and episodes of binge drinking, assessed at all three time points. The criteria of participant selection were based on a previous report ([Whelan et al., 2014](#_ENREF_10)). More details are provided as eMethods. We finally selected 212 19-year-old participants for our study, and only 32 of them underwent rsfMRI data at age 14.

**
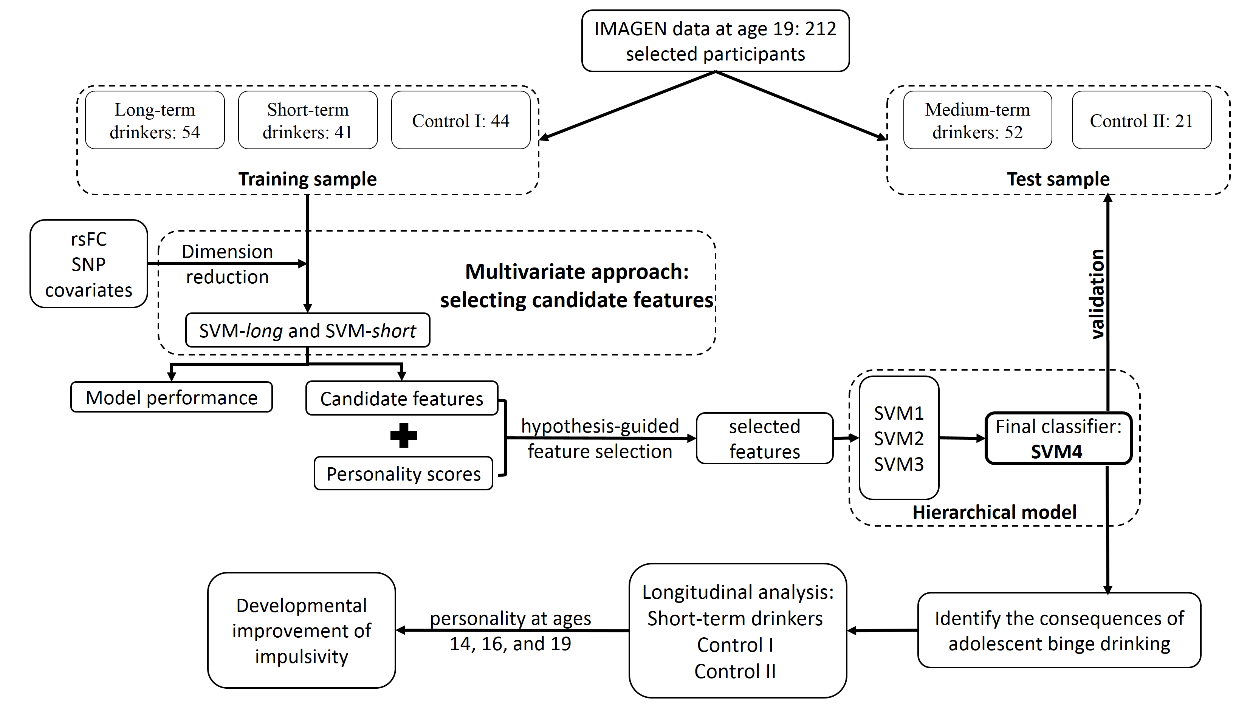
**

# **eFigure 2. Overview of the study design**

We selected 212 participants from the IMAGEN study. We stratified them into 5 groups and further divided them into the training and test samples. Using the training sample, we trained one SVM for the long-term drinkers (SVM-long classifier) and the other SVM for the short-term drinkers (SVM-short classifier). The SVM-long and SVM-short classifiers were used to validate that the domains (rsFC, SNP, covariates) we selected for classification were effective and robust to identify binge drinkers. Next, the candidate features identified by SVM-long and SVM-short, as well as the personality scores, were further selected by hypothesis-guided feature selection procedure. Then the selected features were used as the input of SVM1-3, and the numeric output values of SVM1-3 were used as the input of the final classifier, namely SVM4. We tested the final classifier in the test sample. Finally, we applied a longitudinal analysis on the short-term drinkers and controls (Control I and Control II) to explore the developmental trajectories of improvement in impulsivity.

**
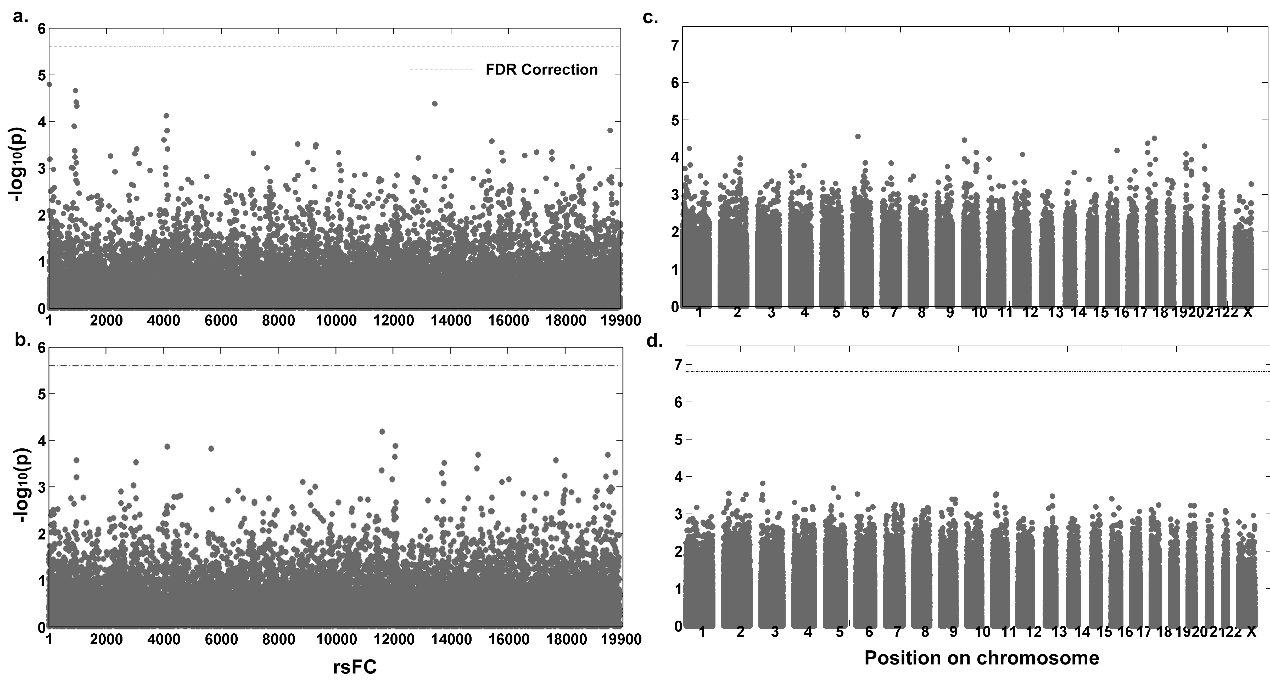
**

**eFigure 3. Manhattan plot of rsFCs and SNPs**

(a), (b) Manhattan plot of the significance level (–log(p)) for all rsFCs compared between different groups of binge drinkers and non-binge controls. The resting-state functional connectivity (rsFC) between each pair of brain regions (19,900 links for 200 atlas-defined brain regions ([Craddock et al., 2012](#_ENREF_2)). The atlas is available at the following website <http://ccraddock.github.io/cluster_roi/atlases.html>. (c), (d) Manhattan plot of the significance level for 324,959 single nucleotide polymorphisms (SNPs). Effects of covariates were always excluded for comparison. Two rows from up to bottom: long-term *vs*. control I, short-term *vs*. control I.

**
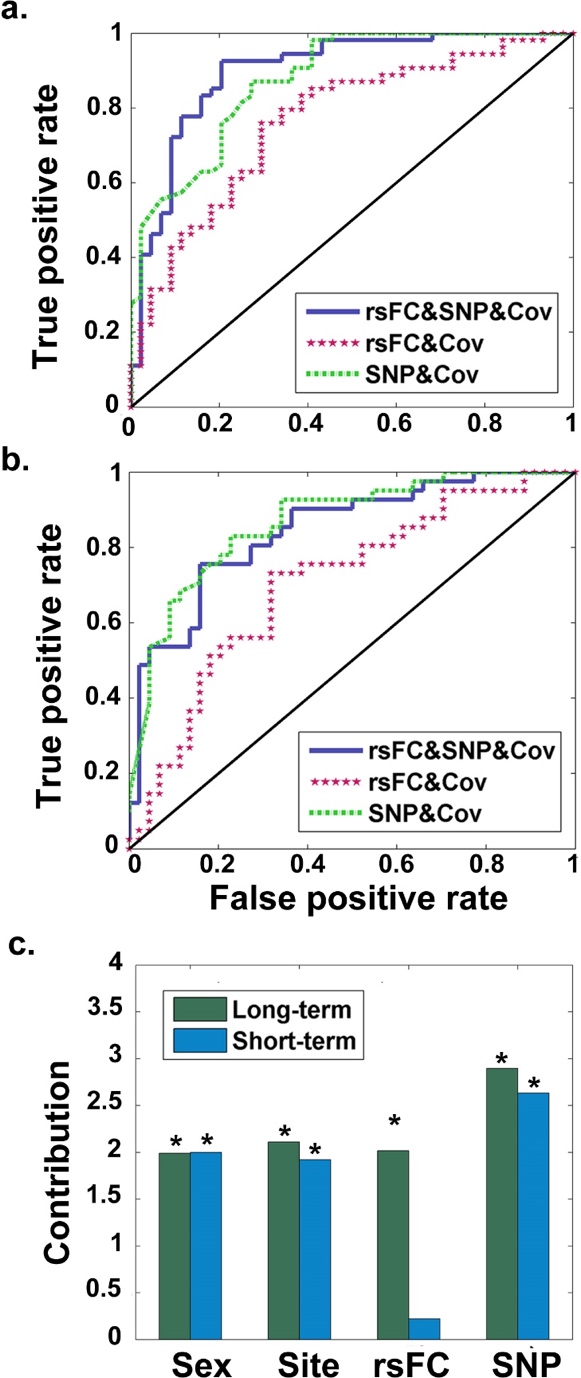
**

**eFigure 4. Performances of different classifiers for binge drinkers and contributions of different features to these models**

Comparison of classification performances based on different features for long- (a) and short-term drinkers (b). rsFC, resting-state functional connectivity; SNP, single polynucleotide polymorphisms; Cov, covariates (including sex and site). (c) Contributions of the features to the classification models. Z-scored net reclassification improvement (NRI) values estimated the reductions in accuracy of classification with each type of feature removed **p* < 0.05, FDR corrected.

**
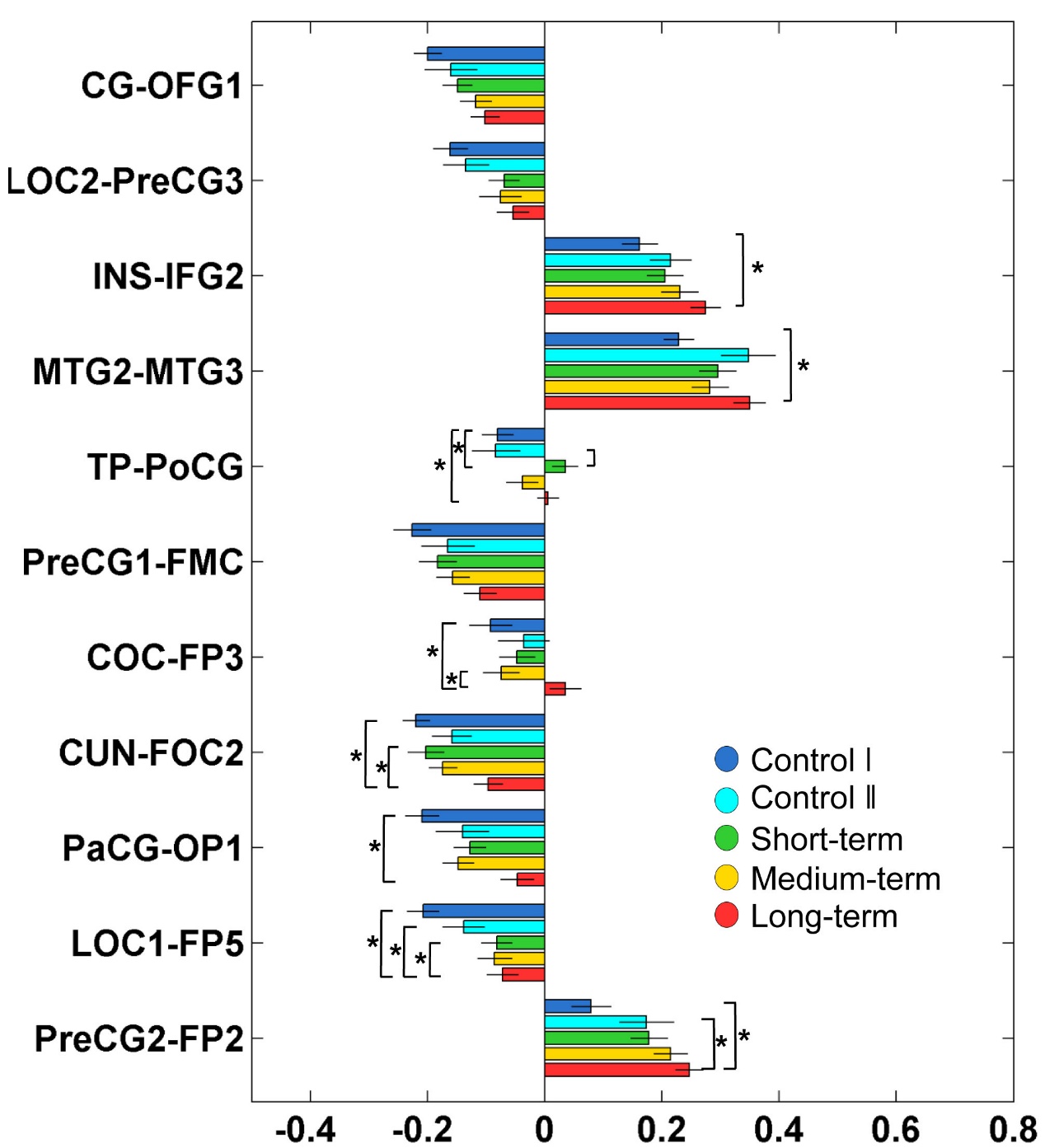
**

**eFigure 5. Comparison on increased rsFCs**

Error bar represents the standard error. **p* < 0.05, FDR corrected. The abbreviations of the brain regions are listed in eTable 3.


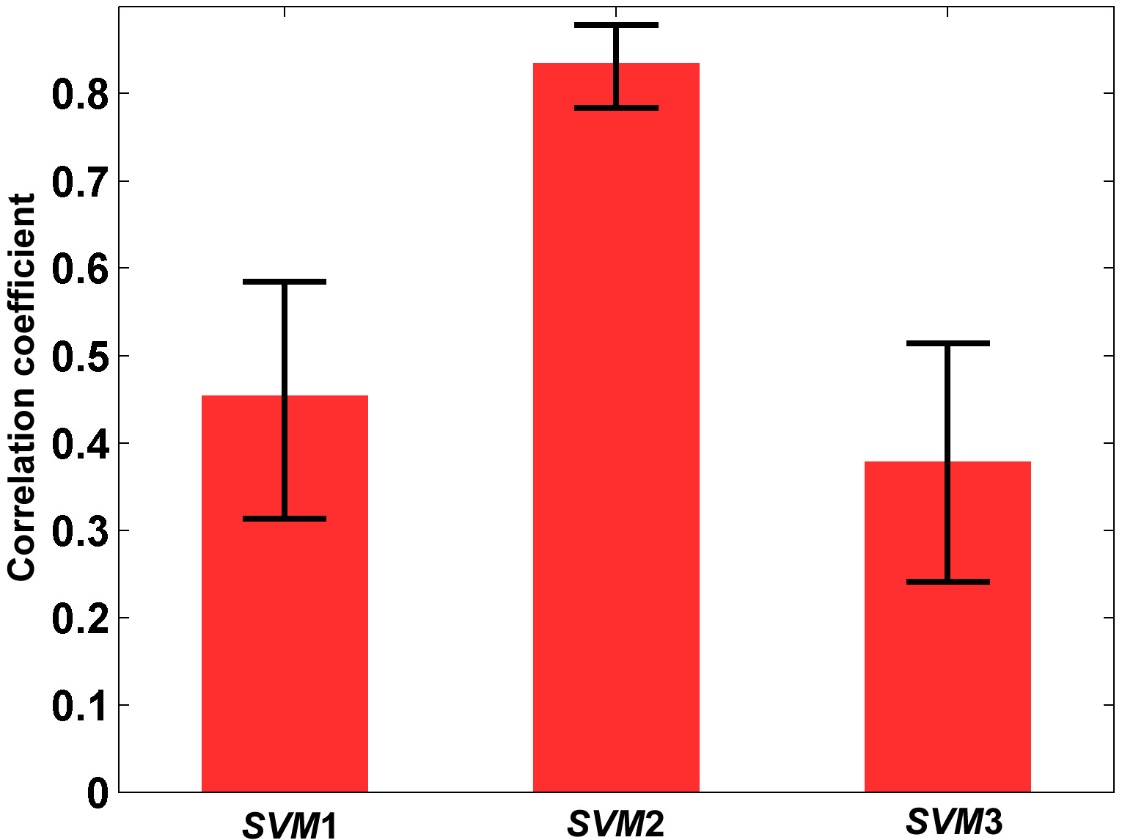


**eFigure 6. Comparison of the contributions by the *SVM*1, *SVM*2, and *SVM*3 to the final model for binge drinking**

Error bar corresponds to the 95% confidence interval resulting from 5000 bootstrap.

**
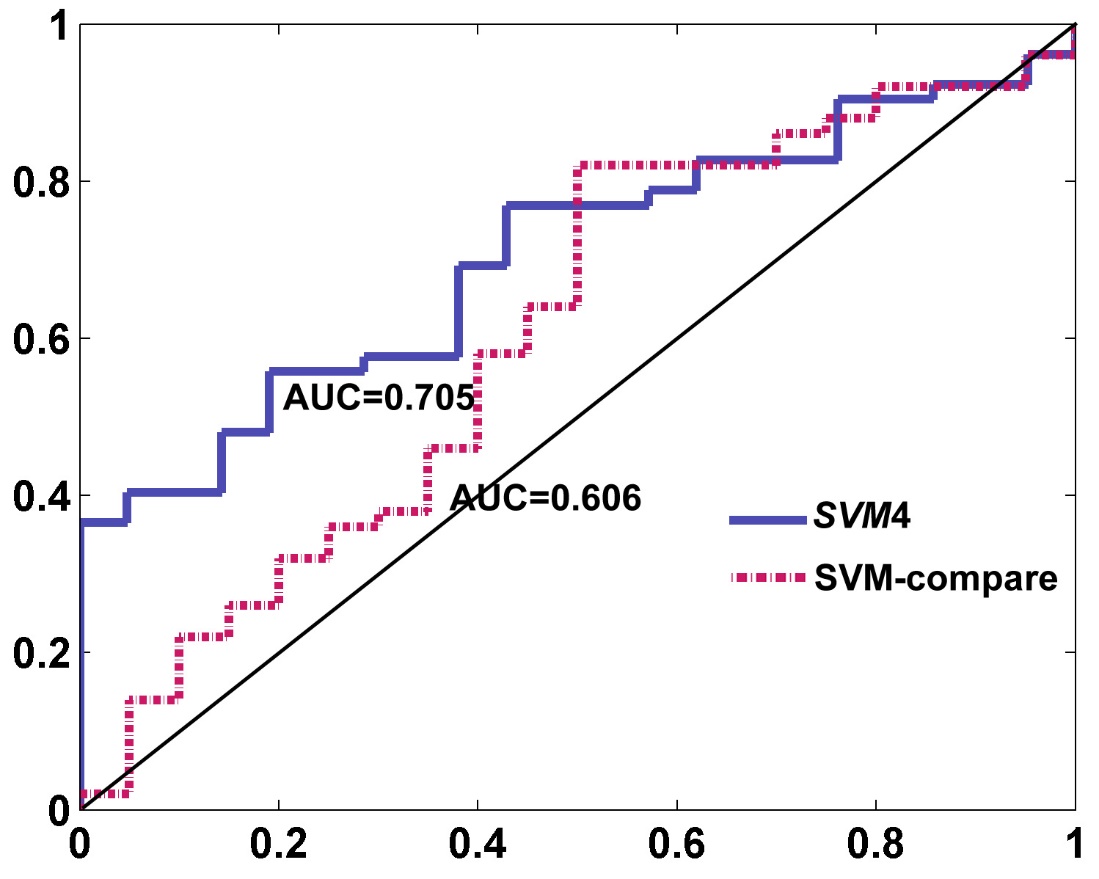
**

**eFigure 7. ROC curves of the *SVM*4 and SVM-comparisons**

We conducted a comparison study by training an SVM named SVM-comparison with a linear kernel using training sample and evaluating its performance on novel test data. The features used in the SVM-comparison were the iFC, dFC, pSNP, and rSNP, as defined in Figure 2 (B), (C) with sex, site, impulsivity, and sensation seeking. The AUC of *SVM*4 is significantly higher than that of SVM-comparison (NRI test, *p* = 0.0372).

**eTable 1. Univariate comparison on cognition tests and personality questionnaires**

| **Cognition** | | |
| --- | --- | --- |
| **Item** | ***p* (long-term vs control I)** | ***p* (short-term vs control I)** |
| **Affective Go-NoGo Latency Negative** | 0.62 | 0.18 |
| **Affective Go-NoGo Latency Positive** | 0.77 | 0.13 |
| **Affective Go-NoGo Omission Negative** | 0.81 | 0.76 |
| **Affective Go-NoGo Omission Positive** | 0.38 | 0.32 |
| **Delay Aversion** | 0.30 | 0.89 |
| **Deliberation Time** | 0.35 | 0.39 |
| **Overall Proportion Bet** | 0.90 | 0.35 |
| **Quality of Decision Making** | 0.62 | 0.88 |
| **Risk Adjustment** | 0.75 | 0.24 |
| **Risk Taking** | 0.89 | 0.27 |
| **Pattern Recognition Memory** | 0.95 | 0.47 |
| **Rapid Visual Processing** | 0.74 | 0.64 |
| **Spatial Working Memory of Errors** | 0.54 | 0.54 |
| **Spatial Working Memory of Strategy** | 0.61 | 0.48 |
| **Overall** | 0.32 | 0.82 |
| **Small** | 0.11 | 0.89 |
| **Medium** | 0.12 | 0.99 |
| **Large** | 0.04 | 0.14 |
| **Mean** | 0.02 | 0.26 |
| **Personality** | | |
| **Item** | ***p* (long-term vs control I)** | ***p* (short-term vs control I)** |
| **Neuroticism** | 0.38 | 0.74 |
| **Extraversion** | 0.03 | 0.02 |
| **Openness** | 0.57 | 0.23 |
| **Conscientiousness** | 0.10 | 0.10 |
| **Agreeableness** | 0.04 | 0.08 |
| **Anxiety** | 0.29 | 0.28 |
| **Negative Thinking** | 0.57 | 0.97 |

**eTable 2. Correlation between the featured rsFC and the long-term binge drinking**

The biserial correlation statistic (*r*) was calculated between each of the rsFCs that were robustly associated with the long-term drinkers during the leave-one-out (LOO) procedure for the SVM-Long (repeatedly selected by over 90% of LOO steps) and the group membership (1 for the long-term drinker; otherwise, 0). The abbreviations of the brain regions were listed in eTable 3.

| **rsFC** | | ***r*** |
| --- | --- | --- |
| **Region 1** | **Region 2** |  |
| PreCG2 | FP2 | 0.39 |
| PaCG | OP1 | 0.38 |
| PreCG4 | LING2 | -0.36 |
| HES1 | LING2 | -0.35 |
| CUN | FOC2 | 0.35 |
| LOC1 | FP5 | 0.34 |
| PHG1 | TFC1 | -0.32 |
| MTG2 | MTG3 | 0.31 |
| PreCG2 | FOC1 | -0.29 |
| INS | IFG2 | 0.28 |
| COC | FP3 | 0.28 |
| CG | OFG1 | 0.28 |
| TP | PoCG | 0.27 |
| PreCG1 | FMC | 0.27 |
| IFG1 | TFC2 | -0.27 |
| LOC2 | PreCG3 | 0.26 |

**eTable 3. Abbreviations for brain regions in this study**

The brain regions in this atlas were labelled by the cluster naming script distributed with the pyClusterROI toolbox (http://ccraddock.github.io/cluster_roi/atlases.html) comparing with Harvard-Oxford Cortical Structural Atlas. For convenience we only list the regions with the abbreviations used in this study.

| **Abbreviation** | **Coordinate** | | | **Name of 'winning region'** | **Found Regions** |
| --- | --- | --- | --- | --- | --- |
| **PaCG** | -7 | 48 | 7 | **Paracingulate Gyrus** | 58% Paracingulate Gyrus, 25% Cingulate Gyrus, anterior division, 2% Superior Frontal Gyrus, 1% Frontal Pole |
| **LOC1** | -50 | -64 | -6 | **Lateral Occipital Cortex** | 51% Lateral Occipital Cortex, inferior division, 17% Middle Temporal Gyrus, temporooccipital part, 14% Inferior Temporal Gyrus, temporooccipital part |
| **PreCG1** | 45 | 11 | 31 | **Precentral Gyrus** | 28% Precentral Gyrus, 17% Middle Frontal Gyrus, 15% Inferior Frontal Gyrus, pars opercularis |
| **CG** | 6 | 45 | 5 | **Cingulate Gyrus** | 56% Cingulate Gyrus, anterior division, 33% Paracingulate Gyrus |
| **TP** | -40 | 8 | -40 | **Temporal Pole** | 76% Temporal Pole |
| **PHG1** | 18 | -32 | -3 | **Parahippocampal Gyrus** | 3% Parahippocampal Gyrus, posterior division, 2% Cingulate Gyrus, posterior division |
| **PreCG2** | -29 | -5 | 57 | **Precentral Gyrus** | 27% Precentral Gyrus, 24% Middle Frontal Gyrus, 14% Superior Frontal Gyrus |
| **FMC** | -1 | 41 | -13 | **Frontal Medial Cortex** | 63% Frontal Medial Cortex, 30% Paracingulate Gyrus, 1% Cingulate Gyrus, anterior division |
| **FOC1** | 44 | 32 | -11 | **Frontal Orbital Cortex** | 45% Frontal Orbital Cortex, 24% Frontal Pole, 5% Inferior Frontal Gyrus, pars triangularis |
| **CUN** | -7 | -85 | 24 | **Cuneal Cortex** | 37% Cuneal Cortex, 4% Occipital Pole, 2% Supracalcarine Cortex, 2% Lateral Occipital Cortex, superoir division |
| **FOC2** | 29 | 26 | -16 | **Frontal Orbital Cortex** | 52% Frontal Orbital Cortex |
| **LOC2** | 46 | -71 | 15 | **Lateral Occipital Cortex** | 42% Lateral Occipital Cortex, inferior division, 30% Lateral Occipital Cortex, superoir division |
| **TFC1** | 36 | -11 | -27 | **Temporal Fusiform Cortex** | 25% Temporal Fusiform Cortex, posterior division, 16% Parahippocampal Gyrus, anterior division, 7% Temporal Fusiform Cortex, anterior division, 2% Inferior Temporal Gyrus, posterior division |
| **PoCG** | -59 | -6 | 25 | **Postcentral Gyrus** | 43% Postcentral Gyrus, 37% Precentral Gyrus |
| **MTG2** | -59 | -47 | -9 | **Middle Temporal Gyrus** | 41% Middle Temporal Gyrus, temporooccipital part, 21% Inferior Temporal Gyrus, temporooccipital part, 8% Middle Temporal Gyrus, posterior division, 2% Inferior Temporal Gyrus, posterior division |
| **PreCG3** | 1 | -22 | 67 | **Precentral Gyrus** | 69% Precentral Gyrus, 4% Juxtapositional Lobule Cortex (formerly Supplementary Motor Cortex) |
| **MTG3** | -57 | -47 | 8 | **Middle Temporal Gyrus** | 33% Middle Temporal Gyrus, temporooccipital part, 19% Supramarginal Gyrus, posterior division, 9% Angular Gyrus, 6% Superior Temporal Gyrus, posterior division, 1% Middle Temporal Gyrus, posterior division |
| **COC** | 55 | 7 | 5 | **Central Opercular Cortex** | 35% Central Opercular Cortex, 26% Precentral Gyrus, 6% Inferior Frontal Gyrus, pars opercularis, 3% Planum Polare, 2% Frontal Operculum Cortex, 1% Temporal Pole |
| **PreCG4** | 0 | -13 | 51 | **Precentral Gyrus** | 32% Precentral Gyrus, 30% Juxtapositional Lobule Cortex (formerly Supplementary Motor Cortex), 6% Cingulate Gyrus, anterior division, 1% Cingulate Gyrus, posterior division |
| **FP2** | -31 | 40 | 33 | **Frontal Pole** | 51% Frontal Pole, 23% Middle Frontal Gyrus, 2% Superior Frontal Gyrus |
| **OP1** | -27 | -91 | 11 | **Occipital Pole** | 38% Occipital Pole, 9% Lateral Occipital Cortex, superoir division, 5% Lateral Occipital Cortex, inferior division |
| **INS** | 40 | -9 | -5 | **Insular Cortex** | 60% Insular Cortex, 9% Planum Polare |
| **IFG1** | -50 | 25 | 8 | **Inferior Frontal Gyrus** | 37% Inferior Frontal Gyrus, pars triangularis, 19% Inferior Frontal Gyrus, pars opercularis, 2% Frontal Operculum Cortex |
| **IFG2** | 51 | 30 | 4 | **Inferior Frontal Gyrus** | 49% Inferior Frontal Gyrus, pars triangularis, 8% Frontal Pole, 2% Inferior Frontal Gyrus, pars opercularis, 1% Frontal Operculum Cortex, 1% Frontal Orbital Cortex |
| **HES1** | -57 | -9 | 4 | **Heschl's Gyrus (includes H1 and H2)** | 22% Heschl's Gyrus (includes H1 and H2), 19% Planum Polare, 12% Central Opercular Cortex, 11% Planum Temporale, 7% Superior Temporal Gyrus, anterior division, 2% Superior Temporal Gyrus, posterior division |
| **OFG1** | -22 | -90 | -13 | **Occipital Fusiform Gyrus** | 29% Occipital Fusiform Gyrus, 19% Occipital Pole, 11% Lateral Occipital Cortex, inferior division, 1% Lingual Gyrus |
| **FP3** | -27 | 54 | 22 | **Frontal Pole** | 83% Frontal Pole |
| **FP5** | -28 | 59 | 4 | **Frontal Pole** | 77% Frontal Pole |
| **LING2** | 8 | -85 | -13 | **Lingual Gyrus** | 32% Lingual Gyrus, 10% Occipital Fusiform Gyrus, 6% Occipital Pole |
| **TFC2** | 30 | 1 | -37 | **Temporal Fusiform Cortex** | 46% Temporal Fusiform Cortex, anterior division, 16% Parahippocampal Gyrus, anterior division, 4% Temporal Fusiform Cortex, posterior division, 2% Temporal Pole |

**eTable 4a. Discriminative SNPs selected by the multivariate model for long-term drinkers**

Chr – Chromosome; Cat—Categories of **r**isk or **p**rotective.

| **SNP** | **Chr** | **Gene** | **Cat** | **SNP** | **Chr** | **Gene** | **Cat** |
| --- | --- | --- | --- | --- | --- | --- | --- |
| rs12760004 | 1 | NA | r | rs3827672 | 9 | SLC31A2 | r |
| rs10889403 | 1 | NA | r | rs10991853 | 9 | AUH | r |
| rs4650278 | 1 | NA | p | rs2165951 | 9 | NA | p |
| rs1395718 | 1 | ZC3H12A | r | rs1451185 | 10 | NA | r |
| rs10799802 | 1 | NA | r | rs7894041 | 10 | NA | r |
| rs2050249 | 1 | NA | p | rs2907554 | 10 | ATRNL1 | p |
| rs12141297 | 1 | USH2A | p | rs725556 | 10 | ATRNL1 | p |
| rs2353187 | 2 | BBS5 | p | rs2148887 | 10 | NA | r |
| rs1495824 | 3 | NA | r | rs2960662 | 10 | ATRNL1 | p |
| rs13084044 | 3 | NA | r | rs1219508 | 10 | NA | r |
| rs2033447 | 3 | RARB | p | rs2139518 | 11 | NA | r |
| rs1396696 | 4 | NA | r | rs10894965 | 11 | NA | r |
| rs17590593 | 4 | ANK2 | p | rs1911723 | 12 | NA | r |
| rs42495 | 5 | SEMA5A | p | rs12313946 | 12 | LOC387867 | p |
| rs185248 | 5 | SEMA5A | p | rs2647955 | 12 | NA | p |
| rs28181 | 5 | SEMA5A | p | rs4275668 | 12 | NA | r |
| rs28180 | 5 | SEMA5A | p | rs4409904 | 12 | NA | p |
| rs7743421 | 6 | NA | r | rs2061589 | 12 | NA | p |
| rs679881 | 6 | NA | r | rs4073984 | 12 | NA | p |
| rs1970456 | 6 | CRISP1 | p | rs11107062 | 12 | NA | r |
| rs10484732 | 6 | NA | p | rs7973014 | 12 | NA | r |
| rs2817318 | 6 | NA | p | rs12434963 | 14 | NA | r |
| rs406541 | 6 | NA | r | rs17451475 | 14 | NA | r |
| rs2209936 | 6 | NA | r | rs4842862 | 15 | NA | r |
| rs2852498 | 6 | NA | r | rs8029847 | 15 | SLC28A1 | r |
| rs6912194 | 6 | NA | p | rs11852308 | 15 | SLC28A1 | r |
| rs9488675 | 6 | NA | p | rs8025045 | 15 | SLC28A1 | r |
| rs1467926 | 6 | NA | p | rs16964032 | 15 | C15orf41 | r |
| rs2770333 | 6 | NA | r | rs10518905 | 15 | C15orf41 | r |
| rs16878696 | 6 | ATXN1 | p | rs154659 | 16 | NA | p |
| rs2479992 | 6 | NA | p | rs7201500 | 16 | A2BP1 | p |
| rs2479996 | 6 | NA | p | rs9284390 | 18 | NA | p |
| rs1007237 | 6 | NA | p | rs7232480 | 18 | NA | p |
| rs926850 | 6 | NA | p | rs4508512 | 18 | NA | p |
| rs1753567 | 6 | NA | r | rs10514150 | 18 | ZNF407 | r |
| rs12530588 | 7 | SEMA3E | r | rs1942581 | 18 | NA | p |
| rs17505926 | 7 | SEMA3E | r | rs11082551 | 18 | ST8SIA5 | p |
| rs7803017 | 7 | NA | p | rs1370911 | 18 | KCTD1 | r |
| rs2538912 | 7 | NA | p | rs1129156 | 19 | MAP3K10 | p |
| rs1453288 | 7 | PKD1L1 | p | rs6010620 | 20 | RTEL1 | p |
| rs6972069 | 7 | COL28A1 | r | rs6122161 | 20 | ZBTB46 | r |
| rs1799022 | 7 | MAGI2 | r | rs2315654 | 20 | ZBTB46 | p |
| rs6943595 | 7 | NA | p | rs6089953 | 20 | RTEL1 | p |
| rs429412 | 8 | NA | r | rs6135562 | 20 | MACROD2 | p |
| rs4529495 | 8 | NA | p | rs6042962 | 20 | MACROD2 | r |
| rs375306 | 8 | NA | r | rs407097 | 20 | MACROD2 | r |
| rs2948998 | 8 | NA | r | rs6079823 | 20 | MACROD2 | r |
| rs10098719 | 8 | MTUS1 | r | rs8130195 | 21 | NA | p |
| rs7026539 | 9 | NA | p | rs1016173 | 21 | NCAM2 | p |
| rs633565 | 9 | GLIS3 | r | rs2070384 | 21 | NA | r |
|  |  |  |  | rs12012447 | X | NA | r |

**eTable 4b. Discriminative FCs selected by the multivariate model for short-term drinkers**

For each FC, the ID of both regions and their coordinates in the atlas were listed.

| **Region1** | **Coordinate** | | | **Region2** | **Coordinate** | | |
| --- | --- | --- | --- | --- | --- | --- | --- |
| 15 | 13 | 15 | -8 | 134 | 23 | -11 | 67 |
| 16 | -50 | -64 | -6 | 183 | -28 | 59 | 4 |
| 17 | 45 | 11 | 31 | 127 | 30 | 20 | 51 |
| 31 | 29 | -53 | 59 | 169 | -42 | 51 | -4 |
| 43 | -40 | 8 | -40 | 185 | 43 | -23 | 15 |
| 44 | 13 | -93 | 1 | 56 | -53 | -49 | 41 |
| 49 | 61 | -19 | -17 | 114 | -27 | -72 | 40 |
| 56 | -53 | -49 | 41 | 167 | -27 | 54 | 22 |
| 56 | -53 | -49 | 41 | 198 | 30 | 1 | -37 |
| 64 | 28 | 1 | 56 | 182 | -9 | 2 | 65 |
| 74 | -45 | 35 | -9 | 149 | -1 | 33 | 44 |
| 75 | 30 | 57 | 14 | 146 | -57 | -9 | 4 |
| 101 | -52 | -2 | -29 | 190 | 15 | -32 | -22 |
| 114 | -27 | -72 | 40 | 143 | 52 | -60 | 0 |
| 123 | 0 | -13 | 51 | 187 | 13 | 23 | 59 |
| 137 | 40 | -9 | -5 | 179 | -14 | -51 | -2 |
| 137 | 40 | -9 | -5 | 187 | 13 | 23 | 59 |
| 138 | 21 | -68 | -12 | 199 | -16 | -28 | -20 |
| 160 | -1 | 22 | -9 | 185 | 43 | -23 | 15 |
| 164 | 51 | 23 | 20 | 183 | -28 | 59 | 4 |

**eTable 4c. Discriminative SNPs selected by the multivariate model for short-term drinkers**

Chr – Chromosome; Cat—Categories of **r**isk or **p**rotective.

| **SNP** | **Chr** | **Gene** | **Cat** | **SNP** | **Chr** | **Gene** | **Cat** |
| --- | --- | --- | --- | --- | --- | --- | --- |
| rs17126889 | 1 | CACHD1 | r | rs4529495 | 8 | NA | p |
| rs10489560 | 1 | NA | r | rs10975884 | 9 | JMJD2C | r |
| rs663669 | 1 | NA | r | rs7857570 | 9 | JMJD2C | r |
| rs13375867 | 1 | SASS6 | r | rs1408118 | 9 | PTPRD | r |
| rs1113500 | 1 | NA | r | rs10961187 | 9 | NA | p |
| rs626737 | 1 | KCND3 | p | rs8192977 | 9 | NA | p |
| rs9426832 | 1 | NA | p | rs1998584 | 9 | NA | p |
| rs1807765 | 1 | FCRL5 | r | rs992457 | 9 | NA | p |
| rs1289002 | 1 | NA | p | rs2165951 | 9 | NA | p |
| rs1290505 | 1 | NA | p | rs12001724 | 9 | NA | r |
| rs858119 | 1 | NA | p | rs868886 | 9 | NA | p |
| rs1773356 | 1 | NA | r | rs4332197 | 9 | NA | p |
| rs10910389 | 1 | SLC35F3 | p | rs10821312 | 9 | LOC158257 | r |
| rs1858409 | 2 | NA | r | rs12379483 | 9 | LOC158257 | r |
| rs17741042 | 2 | NA | p | rs2149981 | 9 | NA | r |
| rs2280516 | 2 | C2orf39 | r | rs10982508 | 9 | TMOD1 | r |
| rs6739975 | 2 | TTC7A | r | rs16914500 | 9 | PALM2 | r |
| rs2163982 | 2 | TTC7A | r | rs10817411 | 9 | NA | r |
| rs9808216 | 2 | NA | r | rs2297181 | 9 | TNC | p |
| rs6716141 | 2 | NA | r | rs2297179 | 9 | TNC | p |
| rs7605051 | 2 | NA | p | rs7865756 | 9 | NA | p |
| rs1011422 | 2 | NA | p | rs4837698 | 9 | NA | p |
| rs11690295 | 2 | NA | r | rs10751920 | 10 | NA | r |
| rs6759275 | 2 | NA | r | rs7072398 | 10 | IL2RA | p |
| rs1561266 | 2 | TTL | r | rs4749926 | 10 | IL2RA | p |
| rs13024113 | 2 | KYNU | p | rs7893981 | 10 | FRMD4A | p |
| rs1515926 | 2 | NA | p | rs10906652 | 10 | NA | r |
| rs2356507 | 2 | NA | p | rs1572202 | 10 | NA | r |
| rs2166512 | 2 | LOC100129028 | p | rs1171594 | 10 | NA | r |
| rs6715752 | 2 | NA | r | rs6586051 | 10 | LOC727923 | r |
| rs10497870 | 2 | NA | r | rs10510045 | 10 | NA | r |
| rs1153452 | 3 | NA | p | rs2296546 | 10 | C10orf46 | r |
| rs6775191 | 3 | NA | p | rs2404076 | 11 | NA | r |
| rs4685048 | 3 | NA | p | rs4433531 | 11 | NA | r |
| rs9881202 | 3 | CAPN7 | r | rs10501007 | 11 | NA | r |
| rs6777812 | 3 | COLQ | r | rs7113764 | 11 | MPPED2 | r |
| rs1604003 | 3 | NA | r | rs1943620 | 11 | NCAM1 | r |
| rs1603999 | 3 | NA | r | rs2574824 | 11 | NCAM1 | r |
| rs9821993 | 3 | NA | p | rs965560 | 11 | NCAM1 | r |
| rs1373605 | 3 | NA | r | rs726601 | 11 | SORL1 | p |
| rs1001689 | 3 | NA | p | rs1503415 | 11 | SORL1 | p |
| rs17345089 | 3 | CLSTN2 | r | rs7949483 | 11 | NA | p |
| rs9810857 | 3 | SLC9A9 | p | rs4073984 | 12 | NA | p |
| rs4679735 | 3 | NA | p | rs4409904 | 12 | NA | p |
| rs4299495 | 3 | NA | p | rs4073042 | 12 | NA | p |
| rs10516262 | 4 | NA | p | rs718314 | 12 | NA | r |
| rs542309 | 4 | NA | r | rs2131159 | 12 | KRT4 | r |
| rs695053 | 4 | NA | r | rs7977082 | 12 | NA | p |
| rs1318557 | 4 | NA | p | rs11109097 | 12 | NA | p |
| rs10516858 | 4 | NA | p | rs114771 | 12 | ANKS1B | r |
| rs9884908 | 4 | NA | r | rs11060894 | 12 | RIMBP2 | r |
| rs6837683 | 4 | NA | p | rs7322971 | 13 | RCBTB1 | p |
| rs10488855 | 4 | NA | p | rs4942848 | 13 | RCBTB1 | p |
| rs2029635 | 4 | NA | r | rs7337571 | 13 | RCBTB1 | p |
| rs10015200 | 4 | NA | r | rs301678 | 13 | NA | r |
| rs12507901 | 4 | NA | r | rs301661 | 13 | NA | r |
| rs10024014 | 4 | GLRA3 | r | rs300307 | 13 | NA | r |
| rs10000456 | 4 | GLRA3 | r | rs300310 | 13 | NA | r |
| rs6419960 | 4 | NA | p | rs1532807 | 13 | NA | r |
| rs2077369 | 5 | NA | r | rs12874899 | 13 | NA | r |
| rs13158156 | 5 | NA | r | rs9592234 | 13 | NA | r |
| rs10755235 | 5 | PARP8 | p | rs9598563 | 13 | NA | r |
| rs282559 | 5 | PARP8 | p | rs9317297 | 13 | NA | r |
| rs3010242 | 5 | NA | r | rs2860230 | 13 | NA | r |
| rs10045155 | 5 | IQGAP2 | p | rs2992154 | 13 | NA | r |
| rs1479210 | 5 | NA | p | rs9570842 | 13 | NA | r |
| rs6862853 | 5 | ATP10B | r | rs1114616 | 13 | NA | p |
| rs9367034 | 6 | NA | p | rs9572072 | 13 | NA | p |
| rs12662776 | 6 | NA | p | rs4885013 | 13 | NA | r |
| rs6903765 | 6 | LRRC16A | r | rs7987156 | 13 | NA | r |
| rs10946785 | 6 | LRRC16A | r | rs593799 | 13 | FARP1 | p |
| rs1980450 | 6 | NA | r | rs2391784 | 13 | NA | p |
| rs876239 | 6 | NA | r | rs7152286 | 14 | NA | p |
| rs2072846 | 6 | SCGN | r | rs11622145 | 14 | GNG2 | r |
| rs2345981 | 6 | NA | p | rs10498440 | 14 | GNG2 | r |
| rs12216537 | 6 | NA | p | rs2236561 | 14 | GNG2 | r |
| rs9384159 | 6 | NA | p | rs17124780 | 14 | GNG2 | r |
| rs3757037 | 6 | NA | p | rs12884801 | 14 | FLVCR2 | r |
| rs1317198 | 7 | NA | p | rs2372236 | 14 | NA | r |
| rs17581713 | 7 | NA | r | rs11159770 | 14 | NA | r |
| rs11764246 | 7 | NA | p | rs10873523 | 14 | NA | p |
| rs11761572 | 7 | NA | p | rs12595109 | 15 | NA | r |
| rs38525 | 7 | JAZF1 | p | rs4077746 | 15 | NA | p |
| rs4723858 | 7 | POU6F2 | r | rs4776758 | 15 | MEGF11 | p |
| rs17659868 | 7 | PKD1L1 | p | rs10852175 | 15 | NA | p |
| rs12668946 | 7 | PKD1L1 | p | rs4787985 | 16 | CACNG3 | r |
| rs7803017 | 7 | NA | p | rs193907 | 16 | ZNF423 | r |
| rs6943595 | 7 | NA | p | rs8051614 | 16 | NA | p |
| rs10281202 | 7 | STRA8 | r | rs9909528 | 17 | SEC14L1 | p |
| rs969356 | 7 | TPK1 | r | rs9284390 | 18 | NA | p |
| rs4549697 | 7 | GALNTL5 | r | rs12373400 | 18 | NA | p |
| rs11772335 | 7 | NA | p | rs11081461 | 18 | NA | p |
| rs6988473 | 8 | NA | r | rs1370911 | 18 | KCTD1 | r |
| rs7002197 | 8 | NA | r | rs3786800 | 19 | ZNF536 | r |
| rs922784 | 8 | NA | r | rs761002 | 20 | NA | r |
| rs767415 | 8 | NA | r | rs6025518 | 20 | RAE1 | r |
| rs2977044 | 8 | JPH1 | p | rs486921 | 20 | RAB22A | r |
| rs926110 | 8 | JPH1 | p | rs6089953 | 20 | RTEL1 | p |
| rs2460221 | 8 | NA | p | rs6010620 | 20 | RTEL1 | p |
| rs10100569 | 8 | CSMD3 | p | rs232381 | 21 | NCAM2 | p |
| rs1006998 | 8 | NA | r | rs10483088 | 21 | NA | p |
| rs1121948 | 8 | NA | p | rs4819850 | 22 | TXNRD2 | r |
| rs11993225 | 8 | NA | r | rs756661 | 22 | TXNRD2 | r |
| rs6471220 | 8 | NA | p | rs2072811 | 22 | NA | p |

**eTable 5. Summary statistics of the increased rsFCs in five groups**

Entries in the first 5 columns indicates ‘mean (standard deviation)’. *P*-values resulted from analysis of variance for rsFC’s.

|  | **Long-term** | **Medium-term** | **Short-term** | **Control II** | **Control I** | ***P*-value** |
| --- | --- | --- | --- | --- | --- | --- |
| **CG-OFG1** | -0.10(0.18) | -0.12(0.17) | -0.15(0.18) | -0.16(0.21) | -0.20(0.16) | 0.101 |
| **LOC2-PreCG3** | -0.05(0.20) | -0.08(0.23) | -0.07(0.19) | -0.13(0.18) | -0.16(0.19) | 0.057 |
| **INS-IFG2** | 0.27(0.19) | 0.23(0.20) | 0.21(0.23) | 0.21(0.16) | 0.16(0.20) | 0.071 |
| **MTG2-MTG3** | 0.35(0.20) | 0.28(0.20) | 0.30(0.23) | 0.35(0.21) | 0.23(0.17) | 0.039 |
| **TP-PoCG** | 0.01(0.13) | -0.04(0.17) | 0.03(0.16) | -0.08(0.19) | -0.08(0.18) | $4.52\times{10}^{-4}$ |
| **PreCG1-FMC** | -0.11(0.21) | -0.16(0.18) | -0.18(0.23) | -0.17(0.21) | -0.23(0.21) | $7.96\times10^{-4}$ |
| **COC-FP3** | 0.04(0.20) | -0.07(0.20) | -0.05(0.22) | -0.04(0.20) | -0.09(0.24) | 0.019 |
| **CUN-FOC2** | -0.10(0.18) | -0.17(0.15) | -0.20(0.22) | -0.16(0.15) | -0.22(0.15) | $7.27\times{10}^{-3}$ |
| **PaCG-OP1** | -0.05(0.21) | -0.15(0.17) | -0.13(0.20) | -0.14(0.21) | -0.21(0.19) | $1.94\times{10}^{-3}$ |
| **LOC1-FP5** | -0.07(0.20) | -0.09(0.19) | -0.08(0.19) | -0.14(0.16) | -0.21(0.18) | $2.82\times{10}^{-3}$ |
| **PreCG2-FP2** | 0.25(0.17) | 0.21(0.18) | 0.18(0.23) | 0.17(0.21) | 0.08(0.22) | $1.61\times{10}^{-3}$ |

**eTable 6. Comparison of classification accuracies in each group by different models**

Classification accuracies of different behavioural groups by different models, including the *SVM1* (based on the sex and the data collection site), the *SVM2* (based on both the increased score of rsFC and the risk SNP for binge), *SVM3* (based on personality), and the *SVM4* (based on the outputs of both the *SVM1* and the *SVM2*).

|  | **Medium-term** | **Control II** |
| --- | --- | --- |
| ***SVM*1** | 65.4% | 57.1% |
| ***SVM*2** | 67.3% | 47.6% |
| ***SVM*3** | 50.0% | 61.9% |
| ***SVM*4** | **76.9%** | **57.1%** |

References

Costa, P. T., & Mccrae, R. R. (1992). Neo PI-R professional manual. *7*(4), 329-345.

Craddock, R. C., James, G. A., Holtzheimer, P. E., Hu, X., & Mayberg, H. S. (2012). A whole brain fMRI atlas generated via spatially constrained spectral clustering. *Human brain mapping, 33*(8), 1914-1928.

Kirby, K. N., Petry, N. M., & Bickel, W. K. (1999). Heroin Addicts Have Higher Discount Rates for Delayed Rewards Than Non-Drug-Using Controls. *Journal of Experimental Psychology: General, 128*(1), 78-87.

Pencina, M. J., Agostino, R. B. D., & Vasan, R. S. (2008). Evaluating the added predictive ability of a new marker: From area under the ROC curve to reclassification and beyond. *Statistics in Medicine, 27*(2), 157-172.

Power, J. D., Barnes, K. A., Snyder, A. Z., Schlaggar, B. L., & Petersen, S. E. (2012). Spurious but systematic correlations in functional connectivity MRI networks arise from subject motion. *Neuroimage, 59*(3), 2142-2154.

Richiardi, J., Altmann, A., Milazzo, A.-C., Chang, C., Chakravarty, M. M., Banaschewski, T., . . . consortium, I. (2015). Correlated gene expression supports synchronous activity in brain networks. *Science (New York, N.Y.), 348*(6240), 1241-1244. doi: 10.1126/science.1255905

Sahakian, B. J., Morris, R. G., Evenden, J. L., Heald, A. E., Levy, R., Philpot, M. P., & Robbins, T. W. (1988). A comparative study of visuospatial memory and learning in Alzheimer-type dementia and Parkinson's disease. *Brain, 111*(3), 695-718.

Schumann, G., Loth, E., Banaschewski, T., Barbot, A., Barker, G., Büchel, C., . . . Gallinat, J. (2010). The IMAGEN study: reinforcement-related behaviour in normal brain function and psychopathology. *Molecular Psychiatry, 15*(12), 1128-1139.

Suykens, J. A. K., & Vandewalle, J. (1999). Least Squares Support Vector Machine Classifiers. *Neural Processing Letters, 9*(3), 293-300.

Whelan, R., Watts, R., Orr, C. A., Althoff, R. R., Artiges, E., Banaschewski, T., . . . Carvalho, F. M. (2014). Neuropsychosocial profiles of current and future adolescent alcohol misusers. *Nature, 512*(7513), 185.

Woicik, P. A., Stewart, S. H., Pihl, R. O., & Conrod, P. (2009). The substance use risk profile scale: A scale measuring traits linked to reinforcement-specific substance use profiles. *Addictive Behaviors, 34*(12), 1042-1055.

**IMAGEN consortium author list**

Lisa Albrecht (Charité), Chris Andrew (IoP), Mercedes Arroyo (Cambridge University), Eric Artiges (INSERM), Semiha Aydin (PTB), Christine Bach (Central Institute of Mental Health), Tobias Banaschewski (Central Institute of Mental Health), Alexis Barbot (Commissariat à l'Energie Atomique), Gareth Barker (IoP), Nathalie Boddaert (INSERM), Arun Bokde (Trinity College Dublin), Zuleima Bricaud (INSERM), Uli Bromberg (University of Hamburg), Ruediger Bruehl (PTB), Christian Büchel (University of Hamburg), Arnaud Cachia (INSERM), Anna Cattrell (IoP), Patricia Conrod (IoP), Patrick Constant (PERTIMM), Jeffrey Dalley (Cambridge University), Benjamin Decideur (Commissariat à l'Energie Atomique), Sylvane Desrivieres (IoP), Tahmine Fadai (University of Hamburg), Herta Flor (Central Institute of Mental Health), Vincent Frouin (Commissariat à l'Energie Atomique), Jürgen Gallinat (Charité), Hugh Garavan (Trinity College Dublin), Fanny Gollier Briand (INSERM), Penny Gowland (University of Nottingham), Bert Heinrichs (Deutsches Referenzzentrum für Ethik), Andreas Heinz (Charité), Nadja Heym (University of Nottingham), Thomas Hübner (Technische Universität Dresden), James Ireland (Delosis), Bernd Ittermann (PTB), Tianye Jia (IoP), Mark Lathrop (CNG), Dirk Lanzerath (Deutsches Referenzzentrum für Ethik), Claire Lawrence (U Nottingham), Hervé Lemaitre (INSERM), Katharina Lüdemann (Charité), Christine Macare (IoP), Catherine Mallik (IoP), Jean-François Mangin (INSERM), Karl Mann (Central Institute of Mental Health), JeanLuc Martinot (INSERM), Eva Mennigen (Technische Universität Dresden ), Fabiana Mesquita de Carvahlo (IoP), Xavier Mignon (PERTIMM), Ruben Miranda (INSERM), Kathrin Müller (Technische Universität Dresden), Frauke Nees (Central Institute of Mental Health), Charlotte Nymberg (IoP), Marie-Laure Paillere (INSERM), Tomas Paus (University of Toronto), Zdenka Pausova (University of Toronto), Jean-Baptiste Poline (Commissariat à l'Energie Atomique), Luise Poustka (Central Institute of Mental Health), Michael Rapp (Charité), Gabriel Robert (IoP), Jan Reuter (Charité), Marcella Rietschel (Central Institute of Mental Health), Stephan Ripke (Technische Universität Dresden), Trevor Robbins (Cambridge University), Sarah Rodehacke (Technische Universität Dresden), John Rogers (Delosis), Alexander Romanowski (Charité), Barbara Ruggeri (IoP), Christine Schmäl (Central Institute of Mental Health), Dirk Schmidt (Technische Universität Dresden), Sophia Schneider (University of Hamburg), MarkGunter Schumann (IoP), Florian Schubert (PTB), Yannick Schwartz (Commissariat à l'Energie Atomique), Michael Smolka (Technische Universität Dresden), Wolfgang Sommer (Central Institute of Mental Health), Rainer Spanagel (Central Institute of Mental Health), Claudia Speiser (GABO:milliarium mbH & Co. KG), Tade Spranger (Deutsches Referenzzentrum für Ethik / Institut of Science and Ethics), Alicia Stedman (University of Nottingham), Sabina Steiner (Central Institute of Mental Health), Dai Stephens (University of Sussex), Nicole Strache (Charité), Andreas Ströhle (Charité), Maren Struve (Central Institute of Mental Health), Naresh Subramaniam (Cambridge University), Lauren Topper (IoP), Walter (Charité), Robert Whelan (University College Dublin), Steve Williams (IoP), Juliana Yacubian (University of Hamburg), Monica Zilbovicius (INSERM), C Peng Wong (IoP), Steven Lubbe (IoP), Lourdes Martinez-Medina (IoP), Alinda Fernandes (IoP), Amir Tahmasebi (University of Toronto)
